# Metagenomic Insights into Pollutants in Biorefinery and Dairy Wastewater: rDNA Dominance and Electricity Generation in Double Chamber Microbial Fuel Cells

**DOI:** 10.3390/bioengineering12010088

**Published:** 2025-01-19

**Authors:** Khaya Pearlman Shabangu, Manimagalay Chetty, Babatunde Femi Bakare

**Affiliations:** 1 Green Engineering & Sustainability Research Group, Department of Chemical Engineering, Faculty of Engineering and the Built Environment, Durban University of Technology, Steve Biko Campus, S3 L3, P.O. Box 1334, Durban 4000, South Africa; chettym@cput.ac.za; 2 Environmental Pollution and Remediation Research Group, Department of Chemical Engineering, Mangosuthu University of Technology, P.O. Box 12363, Jacobs 4062, South Africa; bfemi@mut.ac.za; 3 Department of Chemical Engineering, Cape Peninsula University of Technology, Symphony Way (off Robert Sobukwe Road) Bellville, P.O. Box 1906, Bellville 7535, South Africa

**Keywords:** Bacteriodota, electrical conductivity, Firmicutes, Microbial Fuel Cell, Proteobacteria, salinity, wastewater

## Abstract

This study evaluates the potential of biorefinery and dairy wastewater as substrates for electricity generation in double chamber Microbial Fuel Cells (DCMFC), focusing on their microbial taxonomy and electrochemical viability. Taxonomic analysis using 16S/18S rDNA-targeted DGGE and high-throughput sequencing identified Proteobacteria as dominant in biorefinery biomass, followed by Firmicutes and Bacteriodota. In dairy biomass, Lactobacillus (77.36%) and Clostridium (15.70%) were most prevalent. Biorefinery wastewater exhibited the highest bioelectrochemical viability due to its superior electrical conductivity and salinity, achieving a voltage yield of 65 mV, compared to 75.2 mV from mixed substrates and 1.7 mV from dairy wastewater. Elevated phosphate levels in dairy wastewater inhibited bioelectrochemical processes. This study recommends Biorefinery wastewater as the most suitable purely organic substrate for efficient bioelectricity generation and scaling up of MFCs, emphasising the importance of substrate selection for optimal energy output for practical and commercial viability.

## 1. Introduction

Industrial wastewater, a byproduct of virtually all manufacturing processes, often contains chemical contaminants and reagents, leading to significant environmental pollution and adverse effects on human health when discharged untreated into surface water bodies [1,2]. Dairy wastewater, characterised by discontinuous production processes and significant variability in chemical composition, includes high concentrations of COD, BOD, FOG, nitrogen, and phosphorus, alongside inhibiting cleaning agents [1,3,4,5,6,7,8,9]. While the heterogeneity of dairy wastewater complicates its characterisation, the high-water usage in milk processing generates waste streams with elevated temperatures, variable pH, and sharp fluctuations in contaminant concentrations [4,5,6,7,8,9]. Research on wastewater from other dairy sectors remains limited, necessitating further investigation [4,5,6,7,8,9].

The sugar industry also produces substantial wastewater during seasonal operations, requiring 1500–2000 L of water per ton of cane crushed, generating approximately 1000 L of wastewater [10,11,12,13]. Processing stages, including juice extraction, clarification, evaporation, and crystallisation, contribute to significant wastewater volumes, posing environmental challenges [10,11,12,13]. Addressing these challenges, Microbial Fuel Cell (MFC) technology offers a bioelectrochemical approach for treating complex wastewater substrates while simultaneously generating electricity. MFCs efficiently biodegrade organic pollutants (e.g., COD, BOD, TOC, and TSS) using diverse microbial consortia with unique metabolic capabilities [14]. Certain organic-rich substrates, such as food processing wastewater and swine manure, enhance microbial growth and bioelectrochemical activity [14]. MFC technology not only reduces energy demands and sludge production compared to conventional anaerobic digestion but also produces valuable byproducts [1]. This dual functionality has garnered increasing attention for sustainable wastewater treatment and renewable energy production [1,14,15,16]. However, to commercialise MFC technology, further improvements in performance, efficiency, and scalability are necessary, along with identifying viable inoculants and substrates to enhance bioelectrochemical processes [1,14,15,16].

This study focuses on taxonomically classifying three distinct wastewater sources based on their organic substrate complexity and pollutant strength to assess their viability as electron donors for MFCs. Key wastewater parameters, including electrical conductivity, salinity, total dissolved solids, resistivity, oxidation-reduction potential, and organic pollutant concentrations, were analysed to evaluate their suitability for MFC applications. By characterising pollutant capacities and correlating them with energy generation potential, the study aims to optimise MFC performance and maximise bioenergy production.

The research also explores the thermodynamic relationship between pollutant composition and electricity generation efficiency, using the Gibbs free energy principles to elucidate energy conversion mechanisms in MFCs. Wastewater streams will be inoculated into double-chamber H-type MFCs to assess substrate removal and bioelectricity production, identifying the most reliable anolyte among the studied organic wastewater sources. This novel approach leverages pure industrial and organic wastewater substrates as electrogenic sources, advancing MFC technology for wastewater treatment and renewable energy applications.

This study provides new insights into the taxonomic composition and electrochemical properties of distinct wastewater sources, linking their pollutant profiles to energy generation potential in a double chamber Microbial Fuel Cell (DCMFCs). It introduces a novel application of the Gibbs free energy principles to analyse the thermodynamic relationship between pollutant composition and bioelectricity production efficiency. By leveraging pure industrial and organic wastewater substrates as electrogenic sources, the research highlights the potential of these streams for enhancing DCMFC performance and scalability. This dual focus on wastewater treatment and renewable energy production establishes a foundation for optimising bioelectrochemical processes in real-world applications.

## 2. Materials and Methods

This section outlines the characterisation process for three distinct wastewater sources obtained from two local wastewater treatment plants in Durban, South Africa. The objective of the study is to identify the most suitable wastewater source to serve as a viable Microbial Fuel Cell (MFC) anolyte or electron donor based on its physicochemical parameters. The study is divided into two phases: a 3-month-long characterisation phase focused on the collection and analysis of wastewater samples and microbial consortia from these sources. To ensure optimal operation of the MFC unit, both the physicochemical properties of the wastewater sources and the biocatalysts—bacterial species or active biomass—harvested from the wastewater treatment plants were analysed. The bacterial consortia were classified based on their active genomes and dominant species in each sample population. The three wastewater sources, including mixed stream wastewater, were characterised using bacterial consortium samples collected monthly by grab sampling from the wastewater treatment plants over the 3-month characterisation period. Samples were obtained from two distinct facilities: Tongaat-Hulett Sugar Refinery in Rossburgh and Clover Dairy Plant in Queensburgh, Durban.

**Dairy Wastewater Characterisation**

**Figure 1:**
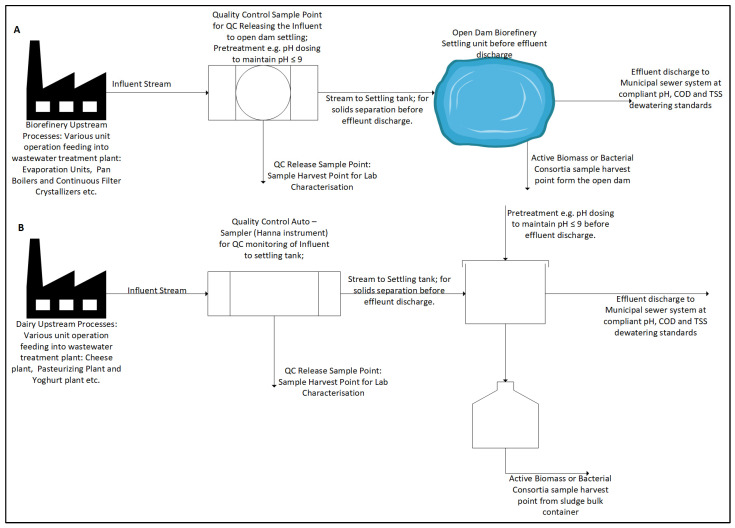
(**A**) Schematic view showing upstream influent discharge plants to the onsite wastewater pretreatment plant and influent sample harvest points for a local biorefinery plant. (**B**) Schematic view for upstream influent discharge plants to onsite Dairy Plant.

Composite samples of dairy wastewater were collected from the influent stream of the Clover Dairy wastewater treatment facility. Effluent was sourced from various sections of the factory, including the pasteurising plant, milk-processing plant, cheese-processing plant, and spillage or wash water. These diverse streams converge into a single effluent that undergoes treatment in a clarifier or settling tank. Key parameters, including total organic carbon (TOC), chemical oxygen demand (COD), electrical conductivity (EC), turbidity, pH, and phosphate levels, were measured at the influent stage before settling. The pH was maintained at approximately 7 before and after settling, prior to dewatering into municipal sewage. Dairy biomass bacterial samples were collected using open-top 2 L buckets to facilitate the settling of denser bacterial masses during storage and inoculation. Samples were preserved at 4 °C in an incubation unit within the chemical engineering laboratory to minimise microbial deviations. A schematic diagram (Figure 1B) illustrates the sampling points for both wastewater and biomass during the dairy wastewater characterisation phase.

**Biorefinery Wastewater Characterisation**

Composite samples of biorefinery wastewater were collected from the influent point of the Tongaat-Hulett Sugar Refinery wastewater treatment facility. Effluent was sourced from various sections of the refinery, including the washing and packaging plant, evaporation pan-boiler processing plant, bleaching and refinery plant, and spillage or wash water. These streams merge into a single influent that feeds into an open ground settling tank for pre-treatment before dewatering. Key parameters, including COD, EC, total dissolved solids (TDS), turbidity, and pH, were monitored as part of quality control before dewatering into municipal receiving bodies. A schematic diagram (Figure 1A) represents the biorefinery wastewater treatment process and sampling points.

**Mixed Wastewater Characterisation**

Dairy wastewater and biorefinery wastewater were combined to create a mixed wastewater source, simulating downstream municipal conditions where effluent streams from various chemical and manufacturing operations converge for further treatment. These mixed streams, characterised by complex organic substrates, were evaluated for their potential as reliable and enduring MFC electron donors or anolytes for bioelectricity production [3,4,5,6,7,8,9,17,18]. To prepare the mixed wastewater samples for experimentation, 50% by volume (*v*/*v*) of dairy and biorefinery wastewater was blended in a 1 L beaker and mixed gently for 24 h at mesophilic temperatures. This blending process was performed immediately using onsite samples from the wastewater treatment plants. The pH was not externally controlled but was allowed to reflect the natural buffering capacity of the combined streams, replicating conditions in municipal receiving bodies. This characterisation phase provides the foundation for assessing the bioelectrochemical potential of these wastewater sources in MFCs. By identifying the most viable electron donor, the study aims to optimise bioelectricity generation and contribute to advancements in wastewater treatment and renewable energy technologies.

### 2.1. Wastewater Sample Characterisation Analytical Approach Before MFC Treatment

The physicochemical characteristics of the three wastewater sources were assessed using standard methods outlined in water and wastewater examination protocols [19,20,21]. Parameters such as temperature (T), pH, oxidation-reduction potential (ORP), conductivity, turbidity, total chemical oxygen demand (tCOD), soluble chemical oxygen demand (sCOD), and total phosphates were evaluated. Measurements of temperature, pH, ORP, electrical conductivity, total dissolved solids (TDS), pHmV, resistivity, and salinity were conducted using a Hanna multiparameter device (HI 9298). Total COD was determined calorimetrically using a Hach DR-3900 spectrophotometer with test vials ranging from 200 to 15,000 mgCOD/L. Total phosphates, total suspended solids (TSS, mg/L), and total organic carbon (TOC, mg/L) were also analysed calorimetrically using the same spectrophotometer. The relationship between TSS and turbidity for the three wastewater samples was empirically established based on guidelines from the Wastewater Engineering Treatment and Reuse handbook by Metcalf and Eddy. Biological oxygen demand (BOD, mg/L) was estimated from tCOD values using a literature-derived COD/BOD ratio of 1:0.6. All measurements were conducted in triplicate and statistically validated at a 95% confidence level to ensure accuracy. For the purposes of accuracy, precision, and reliability, all characterisation sample analysis and in-process analysis during the DCMFC operation were performed in replicates of three.

### 2.2. Biomass Harvesting, Culture Preparation, and Incubation Process Before MFC Inoculation

Biomass mixed cultures were acquired using the grab sampling technique from two local wastewater treatment facilities: Tongaat-Hullet sugar in Rossburg, South Africa, and Clover Dairy wastewater treatment plant in Queensburgh, South Africa. The bacterial samples were collected using open-top 2 L buckets to facilitate settling of denser bacterial masses during storage and inoculation. To preserve the samples and minimise microbiological alterations, they were stored in the chemical engineering laboratory incubation unit at 4 °C. The anoxic microbes were then acclimated and inoculated in 1000 mL blue cap glass bottles to cultivate proficient microbial activity suitable for organic and non-organic removal efficiencies. Following the acclimatization process, the microbes were filtered through appropriate strainers to selectively isolate the desired microbes necessary to enhance the growth rate of electrogene, thereby promoting electron flow within the anodic chamber. After an incubation period of 3 months, skilled technicians from a local bio-analytical consulting laboratory (Inqaba Laboratories—Johannesburg, South Africa) conducted a comprehensive classification of the genomic species to identify bacterial dominance.

### 2.3. Biomass Analytical Characterisation Methods and Instruments

#### 2.3.1. SEM/EDX and Light Preparation

A drop of the suspension was mounted onto an aluminium SEM stub, with double sided carbon tape. These stubs were allowed to air dry and were subsequently gold sputter coated with a Quorum K 150 RES sputter coater. The samples were imaged with a Zeiss Ultra and FEG SEM. Energy Dispersive X-ray (EDX) data were obtained with the Oxford X-Max EDX detector. A drop of the suspension was place on a glass slide with a microscope coverslip. The slides were imaged on a Nikon Eclipse 80i Compound Fluorescent Light microscope. Images were captured with a Nikon DS Fi1 camera. As the electron beam strikes the material, it interacts with the atoms of the various elements.

The atoms have electrons orbiting the nucleus in various layered orbitals. The electrons in these various orbitals have different energies. When the electrons (from the electron beam) strike the electrons in the orbitals, displacement may occur, based on the electron energy difference. The electron from the orbital is displaced by an electron from the beam. The energy liberated from this displacement creates an X-ray. These X-ray energies are listed on the periodic table, for each element, as the Kα; Lα and Mα energies.

A drop of the suspension was applied onto an aluminium SEM stub using double-sided carbon tape. After air drying, the stubs underwent gold sputter coating with a Quorum K 150 RES sputter coater. Imaging of the samples was performed using a Zeiss Ultra and FEG SEM. Energy Dispersive X-ray (EDX) data were collected using the Oxford X-Max EDX detector. Another drop of the suspension was placed on a glass slide and covered with a microscope coverslip. These slides were then examined using the Nikon Eclipse 80i Compound Fluorescent Light microscope. Image acquisition was carried out using a Nikon DS Fi1 camera. As the electron beam interacts with the material, it interacts with the atoms, which have electrons orbiting the nucleus in different layered orbitals. The electrons in these orbitals possess varying energies. Upon collision with the electron beam, electrons in the orbitals may be displaced, resulting in the release of energy in the form of X-rays. The energy of these X-rays depends on the orbital from which the displacement occurred. These X-ray energies, known as Kα, Lα, and Mα energies, are characteristic of each element and are listed on the periodic table.

#### 2.3.2. Biomass rDNA Sequencing and Cloning Procedures

DNA was extracted using the Zymo Research Bacterial and Fungal extraction kit. Full-length 16s PCR was then performed using Forward and reverse universal-tail 16S primers (27F and 1492R) covering variable regions v1 to v9. The resulting Full-length 16s gene amplicons were sequenced on the Sequel-IIe system by PacBio (www.pacb.com). Raw sub-reads were processed through the SMRTlink (v11) Circular Consensus Sequences (CCS) algorithm to produce highly accurate reads *(QV40).* These highly accurate reads were then processed through v-search (https://github.com/torognes/vsearch) and taxonomic information was determined based on QIMME2. The highly accurate sequencing data underwent quality control assessment and taxonomic classification using DADA2 and qiime2, respectively. The processed reads were further analysed using the “create_vsearch_single_sample_pdf_report_pacbio.py” script with the following parameters: input file name (“Clover_Biomass_1432022_M13_bc1004_F--M13_bc1070_R.hifi_reads-filtered-feature-table-asv.tsv”), sample ID (“3_Clover_Biomass_1432022”), cell ID (“220705_Cell1”), and sequencing platform (“16S-QIMME2”).

### 2.4. MFC Operation Design and Conditions

The Microbial Fuel Cell (MFC) system in this study was designed following the specifications outlined by Kim et al. [21]. It consisted of a dual-chamber setup constructed from two 1 L Schutt blue cap bottles. Separating the anode and cathode chambers was a Nafion 115^®^ Proton Exchange Membrane (PEM) from Lyntech, United States, facilitating the transfer of protons (H+) from the anode to the cathode chamber. Carbon-graphite electrodes augmented with copper bi-electrodes were chosen as the electrodes, each with a cross-sectional area of 237 cm^2^. These electrodes, 9 mm in diameter, were mounted on 3 cm diameter flat copper strips in both the anode and cathode chambers. Copper wires were used to connect the electrodes to an external circuit, enabling the transfer of electrons produced from the oxidation of organic matter in the anode chamber to the cathode chamber. For experimental runs and characterisation operations in the Double Chamber Microbial Fuel Cell (DCMFC), a DC/AC load resistor box by LE Lorenzo was employed, with a working range of 1.3 KΩ, consistent with previous studies by Logan et al. [1,14,15,16] and Qing et al. [20]. The cathode compartment was filled with an organic substrate combined with a phosphate-buffer solution, serving as an electron acceptor, and subjected to slow oxygen sparging enhancing the catholyte strength in terms of its composition.

**Figure 2:**
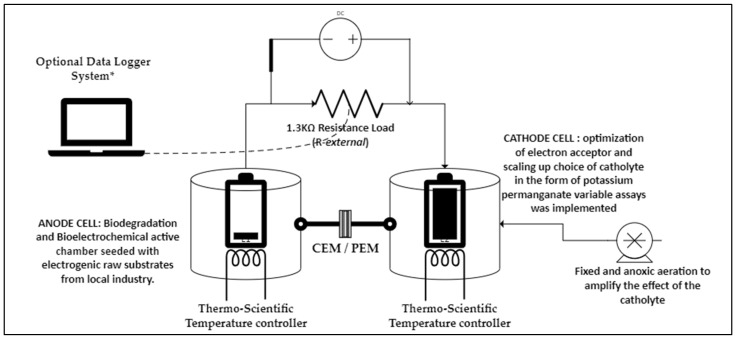
Experimental setup for the DCMFC benchtop for this study, created in CardWorx Professional [22,23]. * Refers to an optional data logger system. The process can be conducted with or without a data logger unit.

A realistic view of the lab-scale DCMFC unit and its components during bench-top operation is illustrated in Figure S4 of the supplementary section. MFC performance was monitored by measuring electrical current (I) and voltage (V) using a multi-meter every 24 h over a 7-day incubation period. The relationship between current and voltage was determined by the equation I = V/R_ext_, with R representing the resistance of an external resistor set to 1.3 KΩ. The connection of the two electrodes via connection terminals, both negative and positive, was completed by a professional electrical technologist after calibrating each electrode for internal resistance and overall load within the system. Figure 2 underneath presents the experimental layout that was utilised for the benchtop DCMFC unit throughout the study. Moreso, the performance monitoring parameters for assessing the overall electrochemical efficacy of the MFC were established based on these parameters and their corresponding models.

(1) I (mA)=VRext

where R_ext_ is the external resistance that was capped at 1300 Ω.V (mV) is the voltage capacity, closed circuit voltage of the MFC unit. While the current is calculated from Ohms law according to Logan et al. [14,15].

(2) $$P\;(mW)=IE_{cell}$$

where P (mW) is the overall power of the MFC unit. Normally, the voltage is measured across a fixed external resistor (R_ext_).

(3) $$P_{anode}\;(mW)=\frac{E_{cell}^{0}}{A_{andoe}R_{ext}}$$

The power output is usually normalised to the projected anode surface area because the anode is where the biological reactions transpire according to Logan et al. [14] and Park et al. [19].

### 2.5. Advanced Statistical Analysis and R Statistical Software

All data obtained for the current study were statistically analysed by calculating the mean, standard deviation (SD), and range. Equations (4) and (5) were used to calculate the mean and SD, respectively.

(4) $$\bar{x}=\frac{\Sigma X}{n}$$

where $\bar{x}$ is the sample mean, and X is the value of each sample; [20,21]. And the sample standard deviation as:

(5) $$SD=\frac{\sqrt{{\left(X-\bar{x}\right)}^{2}}}{n-1}$$

where x is the mean, X is the numerical value of each sample, and n is the total number of samples analysed. The Coefficient of Variation (CV) commonly referred to the ration of standard deviation presented in Equation (6) on the sample mean average as presented in Equation (4), is calculated per following empirical model:

(6) $$CV=\left(\frac{SD}{\bar{x}}\right)*100\%$$

where *SD* = sample standard deviation and $\bar{x}$ = mean for the sample population.

(7) $$\left(\%\right)Removal\;Efficiency\;(Y)=\frac{C_{initial}-C_{final}}{C_{initial}}$$

where Y (%) is the removal efficiency in terms of turbidity, total organic carbon, and/or orthophosphates; C_initial_ and C_final_ are the initial and final concentrations of the targeted contaminants, respectively.

Advanced R Statistical software was utilised for the statistical analysis and interpretation of the raw data collected from the wastewater treatment plant for characterisation purposes. RStudio is an integrated development environment (IDE) for R, providing tools for data analysis, statistical modelling, and visualization. The precise citation details of the model that was use in this study is: Posit, PBC. (2022). RStudio (Version 2022.07.2+576) [Computer software]. Retrieved from https://posit.co/download/rstudio. Specifically, all characterisation runs were conducted entirely independent of the DCMFC operation. Following completion of the wastewater characterisation sequence, the DCMFC trial run were executed to ascertain the potential viable wastewater source as a reliable anolyte in the MFC unit. This analysis underpinned the relevance and significance of various statistical parameters, including median, mean, minimum, and maximum values derived from the raw data tables, as summarised in the correlation plots presented in the results section. To derive a graphical statistical layout for the described approach using R statistical software, one created visualisation such as bar plots, scatter plots, and box plots to represent the statistical analysis and results obtained from Tables S1 and S2, presented in the Supplementary section.

**Figure 3:**
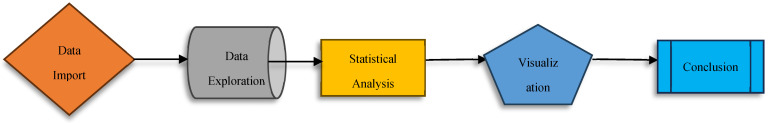
Statistical methodology approach in R-software [14,24,25].

A brief outline of the statistical method using R entailed the following summed up sequence of steps: Importing the experimental characterisation data from into R as data frame. Exploration of the data to understand its structure, summary statistics, and distribution of variables. Conducting a Welch’s *t*-test to compare mean average values between different wastewater streams. Calculate *p*-values, t-values, 95% confidence intervals, and sample mean differences. The calculation of the coefficient of variation (CV) for each parameter was performed subsequently. Essentially, the creation of the bar plots to visualise mean average values of different parameters for each wastewater stream was instigated. Hence, one generated scatter plot to depict the relationship between the organic strength pollutant parameters, such as TOC and COD to list a few. Then, construction of the box plots to display the distribution of the experimental data and identify outliers was carried out. Lastly, it is imperative that one summarises the statistical findings, including significant differences between wastewater streams, relationships between variables, and variability observed. The clear interpretation of the results in the context of the research objectives and implications for bioelectricity generation is well articulated in the next section of this study. Figure 3 summarizes the statistical sequence that was carried out in R-studio statistical analysis software. This was performed for the effective data analyses and presentation of the statistical outcomes from the comparison of the three wastewater streams, and its efficiency for bioelectricity generation as viable electrogenic complex wastewater sources.

## 3. Results and Discussions

### 3.1. Statistical Classification of the Three Wastewater Streams Used in This by Welch Student t-Test and ANOVA Mean Average Test: Biorefinery Wastewater—TH; Dairy Wastewater—(CL) and MIXED-STREAM—(MX)

**Figure 4:**
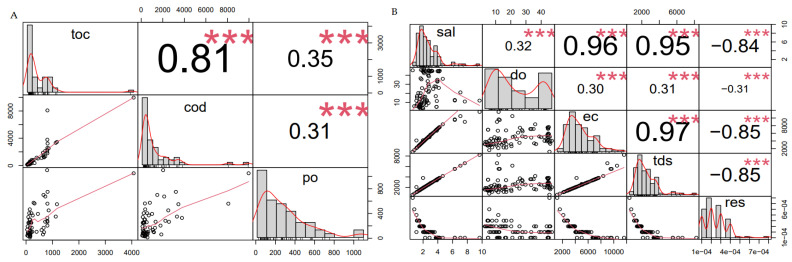
(**A**): Correlation chart from R, for chemical/organic parameters. (**B**): Correlation chart from R, for pysicochemical parameters on the wastewater streams.

Figure 4A,B presents the correlation charts that were performed in R Statistical software as part of the student’s *t*-test method with 95% confidence levels in comparison of both the physico and organic industrial wastewater complex substrates. Figure 4A,B, presents a statistical correlation analysis of key physicochemical parameters related to wastewater quality and their potential implications for bioelectricity production in Microbial Fuel Cells (MFCs). Panel A focuses on the relationships among total organic carbon (TOC), chemical oxygen demand (COD), and phosphates (${PO}_{4}^{3-}$), while Panel B highlights correlations among salinity (Sal), dissolved oxygen (DO), electrical conductivity (EC), total dissolved solids (TDS), and resistivity (Res). Significant correlations are indicated by asterisks (***), and the red regression lines demonstrate linear relationships. In R statistical analysis, the number of asterisks next to a *p*-value in the output of a statistical test or regression summary indicates the level of statistical significance. These asterisks correspond to specific thresholds for the *p*-value: Asterix (***) Indicates a very high level of significance (*p* < 0.001).

Figure 4A shows TOC, COD, and Phosphates: TOC and COD (r = 0.81, *p* < 0.001): A strong positive correlation suggests that TOC levels are proportional to COD, indicating that wastewater with higher organic carbon content tends to have higher chemical oxygen demand. This relationship highlights the potential of TOC-rich wastewater as a substrate for MFCs, as both TOC and COD serve as indicators of available organic matter for microbial metabolism and subsequent electricity generation. TOC and PO (r = 0.35, *p* < 0.001): A weaker positive correlation between TOC and phosphate concentrations suggests that while phosphates are present in wastewater, they do not scale proportionally with organic carbon content. Phosphates, as a nutrient source, could enhance microbial growth but are less directly linked to bioelectricity production compared to organic carbon. COD and $({PO}_{4}^{3-}$), (r = 0.31, *p* < 0.001): Like TOC and $({PO}_{4}^{3-}$), the weak correlation suggests that COD is only moderately related to phosphate levels. This implies that phosphate concentrations may not directly limit or drive COD biodegradation during MFC operations.

Figure 4B shows Salinity, DO, EC, TDS, and Resistivity: Salinity and EC (r = 0.96, *p* < 0.001) and Salinity and TDS (r = 0.95, *p* < 0.001): These strong positive correlations indicate that higher salinity corresponds to increased electrical conductivity and dissolved solids, which are critical parameters for ion transport within the MFC. Elevated salinity and conductivity enhance the ionic strength of the electrolyte, improving electron flow and energy recovery efficiency. Salinity and Resistivity (r = −0.84, *p* < 0.001): The strong negative correlation between salinity and resistivity underscores the inverse relationship, as higher salinity reduces resistivity. This is advantageous for MFC performance, as lower resistivity minimises internal resistance and improves power output. EC and TDS (r = 0.97, *p* < 0.001): The nearly perfect correlation suggests that TDS predominantly governs the electrical conductivity of wastewater. This reinforces the importance of TDS as a key parameter for assessing the suitability of wastewater as an MFC substrate. DO and other parameters (weak correlations): The relatively weak relationships between dissolved oxygen and other parameters suggest limited dependence of DO on salinity, conductivity, or TDS. Since MFCs operate in anoxic conditions, the influence of DO on bioelectricity production may be minimal compared to other physicochemical factors.

Implications for bioelectricity production in DCMFCs: Strong correlations between TOC and COD confirm the potential of these parameters as predictors of organic substrate availability for microbial metabolism. Higher TOC and COD levels are desirable for enhanced electricity generation. Strong correlations among salinity, EC, and TDS highlight the importance of ionic strength in optimising electron transfer and reducing internal resistance in the MFC [19,20,21,26,27,28,29,30,31]. Wastewater with higher salinity and conductivity is better suited for MFC applications. Phosphate levels, though weakly correlated with TOC and COD, remain essential for microbial growth and activity, indirectly supporting bioelectricity production [32,33,34,35,36,37]. The inverse relationship between salinity and resistivity emphasises the need for low-resistivity substrates to maximise MFC efficiency. Overall, this statistical analysis underscores the critical role of organic matter (TOC, COD) and electrolyte conductivity (salinity, EC, TDS) in determining the feasibility of wastewater as an MFC substrate for bioelectricity production.

Tables S1 and S2 in the Supplementary section present the Welch Student’s *t*-test methods used to statistically validate significant differences between the mean average values sourced from the three wastewater streams studied: biorefinery, dairy, and mixed 50% (*v*/*v*) (biorefinery-dairy) wastewater streams. The Welch Student’s t-test analysis was conducted to compare the basic physicochemical parameters commonly referred to as pollutant strengths or complex substrate compositions in industrial wastewater [1,14,15,16,24,25,38]. The objective is to discern significant differences among these wastewater streams and identify the stream with higher and more statistically significant pollutants as a viable source of electrons and proper electron donor or anolyte for a bioelectrochemical technology process to generate bioelectricity in a lab-scale Microbial Fuel Cell (MFC). Precisely, the mean average statistical significances presented in Tables S1 and S2 are derived from thorough statistical analysis using the Welch two-sample Student’s t-test method and 95% confidence level comparison between all wastewater streams for physicochemical parameters [1,14,24,25,38]. This analysis presents mean average values, *p*-values, t-values, 95% confidence interval levels, and sample mean differences when pairing biorefinery-mixed wastewater, dairy-biorefinery, and dairy-mixed wastewater streams. A concluding statement or overview of the statistical regression under the empirical hypothesis that the true difference in mean average values between sample groups listed above for both organic and physicochemical parameters is not equal to zero [25]. 

For most parameters, the *p*-values, discussed based on a set threshold value of 1.02 × 10^−13^ *p* ≤ 0.05, showed strong significance when observed between all groups, as presented in Tables S1 and S2. Additionally, the coefficient of variation (CV), a statistical measure of the dispersion of data points in a data series around the mean, was analysed. The coefficient of variation represents the ratio of the standard deviation to the mean, making it a useful statistic for comparing the degree of variation from one data series to another, even if the means differ drastically from one another [16,24,25]. Based on the findings in Tables S1 and S2, a general hypothesis is proposed that the magnitude of variability of the paired groups’ data was on average low, ranging from 0.43 ≤ CV ≤ 1.473. These low values suggest a very solid observation that the magnitude of the standard deviation to the mean value shown by the CV is minimal. This implies that the average sample data are very close to the mean average value, essentially presenting very small deviations or variability from the mean average data value. Empirically, the data demonstrate a strong confidence that the sample populations for all the groups are not dispersed far from the mean values. Consequently, these values can be reliably used to simulate the chemical or organic matter contained in these complex raw water substrates to predict the amount of bioenergy that can be produced in the form of bioelectricity.

Table S3 in the Supplementary section, presents the Analysis of Variation (ANOVA) between sample groups: biorefinery-dairy; biorefinery-mixed wastewater and dairy- mixed wastewater. This test was performed precisely for the physicochemical parameters: Salinity, Dissolved Oxygen, Electrical Conductivity, Total Dissolved Solids, and Resistivity. A Tukey multiple comparisons of mean average values approach was adopted according to Mutombo et al. [24]. The findings in Table S3 support the statistical hypothesis, indicating a 95% confidence interval for the validity and dispersion of the data based on the sample mean for each population group, be as it may for dairy wastewater, biorefinery wastewater, and the mixed raw wastewater organic substrates. The values presented in Table S3 can be attained at any point of the wastewater treatment plant and any time of the week or process operation. These physicochemical parameter values confirm the conductivity and suitability of all three wastewater organic substrates as anolytes and active electron donors, highlighting their potential as reliable and sustainable sources of bioelectricity in bioelectrochemical systems (BES) precisely the DCMFC system for this study [8,9]. The overall observation of the *p*-value across the comparison groups was 0.000 ≤ *p* ≤ 0.166. This *p*-value presents strong significance of the organic strength of these raw organic industrial wastewater substrates [1,14,16,24,25,38].

### 3.2. Taxonomy and Characterisation of the 3-Complex Substrates Based on Organic Constituents as Viable Electron Donor Before MFC Treatment Stage

#### 3.2.1. Total Organic Carbon (TOC) vs. Chemical Oxygen Demand (COD) Profile for All Wastewater Streams Harvested from the Wastewater Treatment Plant

**Figure 5:**
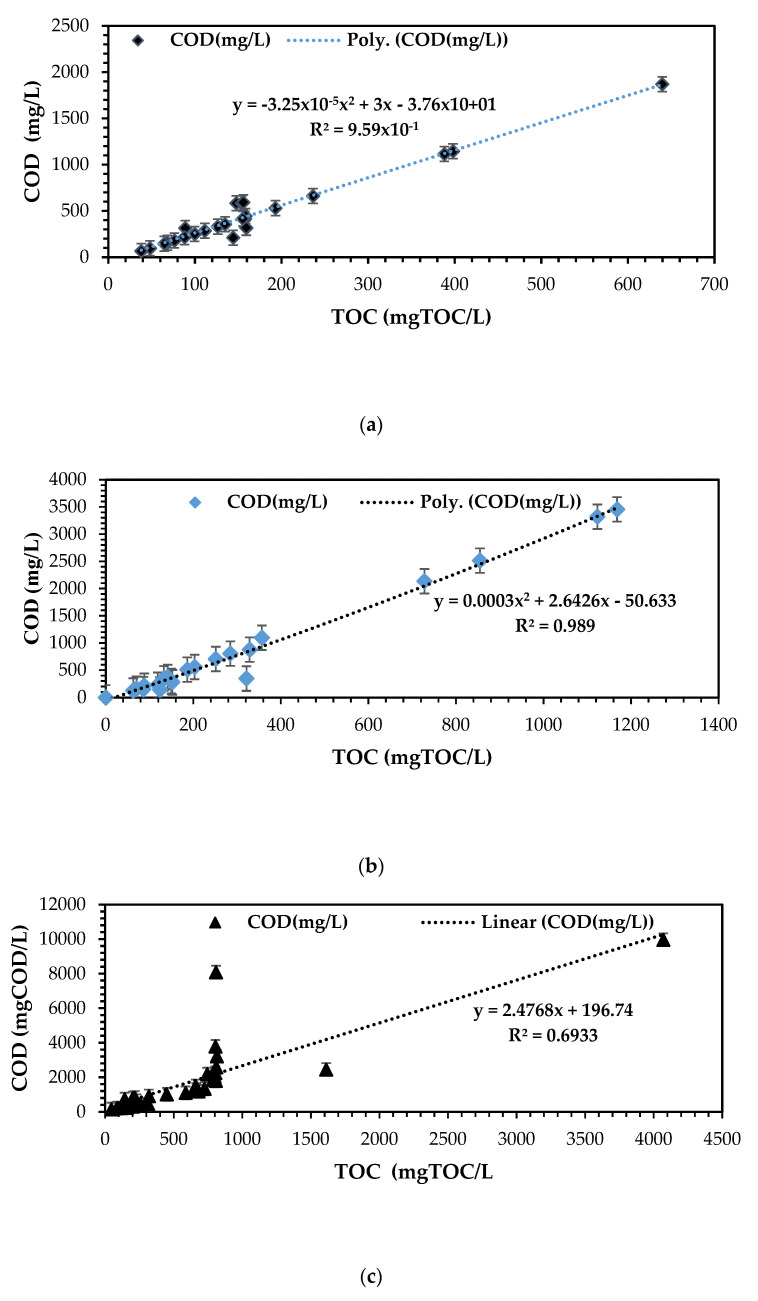
TOC vs. COD for dairy wastewater. (**a**) Dairy wastewater. (**b**) Biorefinery wastewater. (**c**) Mixed wastewater.

Figure 5a–c scientifically proves the strong correlation between total organic carbon and chemical oxygen demand as per the principle of biochemistry and wet chemistry for raw organic substrates principally carbon enriched sources. The literature has reported that a correlation between TOC and COD is normally depicted in the form of linear empirical model [19,20,21,39]:

(8) $$Total\;Organic\;Carbon\;\left(TOC\right)\left(\frac{mgTOC}{L}\right)=\;\frac{COD\;\left(\frac{mgCOD}{L}\right)+49.2}{3}$$

Total organic carbon (TOC) and chemical oxygen demand (COD) monitoring serve as established standards for assessing water quality, both at the point of water injection and treatment [39]. While TOC measures the level of organic molecules or pollutants in water analytically, COD provides a measurement relating to virtually all degradable carbon present in wastewater, [40]. Ongoing advances in the precision and sensitivity of monitoring technologies play a pivotal role in understanding this emerging challenge [9,19,20,21,26,39]. The findings of this study clearly demonstrate a linear relationship between TOC and COD, with a strong correlation indicated by the root mean factor for the three different sources of organic substrates. For instance, Figure 5a illustrates the dairy wastewater effluent’s R^2^ mean factor of 0.989, signifying a strong correlation between TOC and COD. An empirical polynomial correlation model of y = 0.0003x^2^ + 2.6426x − 50.633 was derived to depict the significant relationship between TOC and COD. This model aligns with the theoretical principle that the TOC magnitude in organic industrial wastewater is typically approximately half the composition of COD within the system [19,39], a principle validated by the study. Additionally, dairy wastewater presents a significant amount of chemical energy that could be biodegradable in the form of cATP and chemically converted into bioenergy, provided there are adequate active microorganisms to carry out the biodegradation and electron donation process in bioelectrochemistry within a suitable bioelectrochemical technology such as the MFC system.

Figure 5b for biorefinery wastewater effluent attained a R^2^ mean factor of 0.9588 strong correlation significance between TOC and COD. An empirical polynomial correlation model of y = −3 × 10^−5^x^2^ + 2.9965x − 37.649 was attained in relation to the strong significance between TOC and COD organic substrates. This model is in line with the theoretical principle that the magnitude of the TOC contained in the complex organic industrial wastewater is always almost half the of the composition of COD within the system [19,20,21,39]. The biorefinery stream articulated a good source of chemical energy and potential for being a reliable source of electron donor in the DCMFC system for sustainable energy production. The complex waste matter will be simply harnessed and converted into bioelectricity through the bioelectrochemical principle reported by Logan et al. [1,14,15,16,20,25,38,41].

Figure 5c presented the mixed stream 50% (*v*/*v*) (dairy and biorefinery wastewater effluent systems). The results attained relates to a R^2^ mean factor of 0.693 good correlation significance between TOC and COD. This aspect is scientifically validated by the nature of this wastewater stream. An empirical linear correlation model of y = 2.4768x + 196.74 was attained in relation to the strong significance between TOC and COD organic substrates. This aspect proves that a fraction of COD content in the raw wastewater source correlates to a certain magnitude to the organic composition of TOC in the effluent stream [19,20,21,26,39]. This model is in line with the principle that the magnitude of the TOC contained in the organic substrate is always almost half the of the composition of COD [19].

#### 3.2.2. Salinity Taxonomical Classification and Profile for All Wastewater Streams, Harvested from the Wastewater Treatment Plant

The resistivity of wastewater reflects the ability of wastewater to efficiently resist an electrical current [19,20,21] in a typical MFC system as a viable anolyte. This section scrutinises resistivity of the three wastewater sources and how it relates to viable MFC applications. There is a close link between conductivity and resistivity [19]. While conductivity is a measurement of how well electrical current can flow through wastewater, resistivity is a measurement of how well wastewater can resist electricity flow [19]. An increase in salinity results in an increase in conductivity due to dissolved salts that tend to exude an electrical current [19,21].

**Figure 6:**
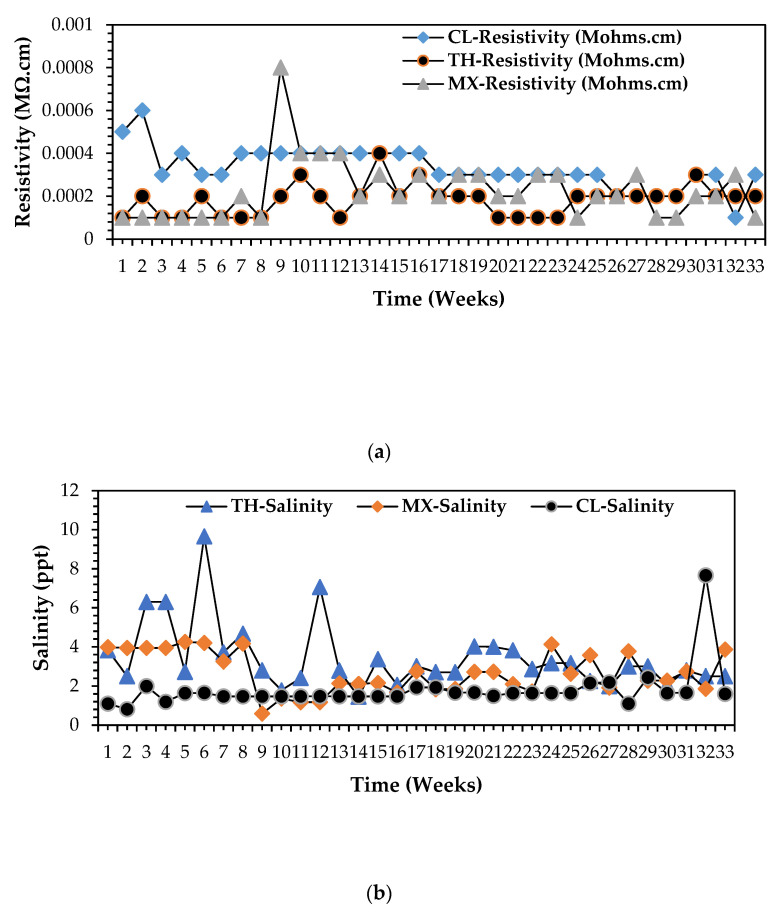
(**a**) Resistivity comparisons against all streams. (**b**) Salinity comparisons, against all streams.

The findings presented in the Supplementary section by Figure S1a shows a strong opposing mechanism between salinity and resistivity, as stated above. A clear characteristic potential is observed between the above streams that dairy wastewater exudes high resistivities and moderate salinity concentrations, referenced in Figure S1a. The highest salinity of 8 ppt and resistivity of 0.0006 mΩ.cm was captured in the dairy wastewater stream referenced by Figure S1a. Contrary, Figure S1b presented high salinity assays of 10 ppt and low resistivities of 0.0001 mΩ.cm. The biorefinery stream exudes the characteristics of a viable anolyte or electron donor in the MFC operation for the production electrical energy, according to Eddie and Metcalf [19]. The concept is validated further by the comparison curves in Figure 6a,b, respectively. Figure 6b presents the high salinity content of a biorefinery wastewater stream as compared to the rest; likewise, Figure 6a clearly shows that biorefinery wastewater poses the least resistance in terms of electron flow sequence with the circuit, hence less resistivity capacity. Precisely, biorefinery seems as a recommendable MFC anolyte for optimised and scaled up MFC electricity production and practical applications with non-exogenous organic complex substrates source.

#### 3.2.3. ORP Taxonomical Profiles for All Streams Harvested from Wastewater Treatment Plant

**Figure 7:**
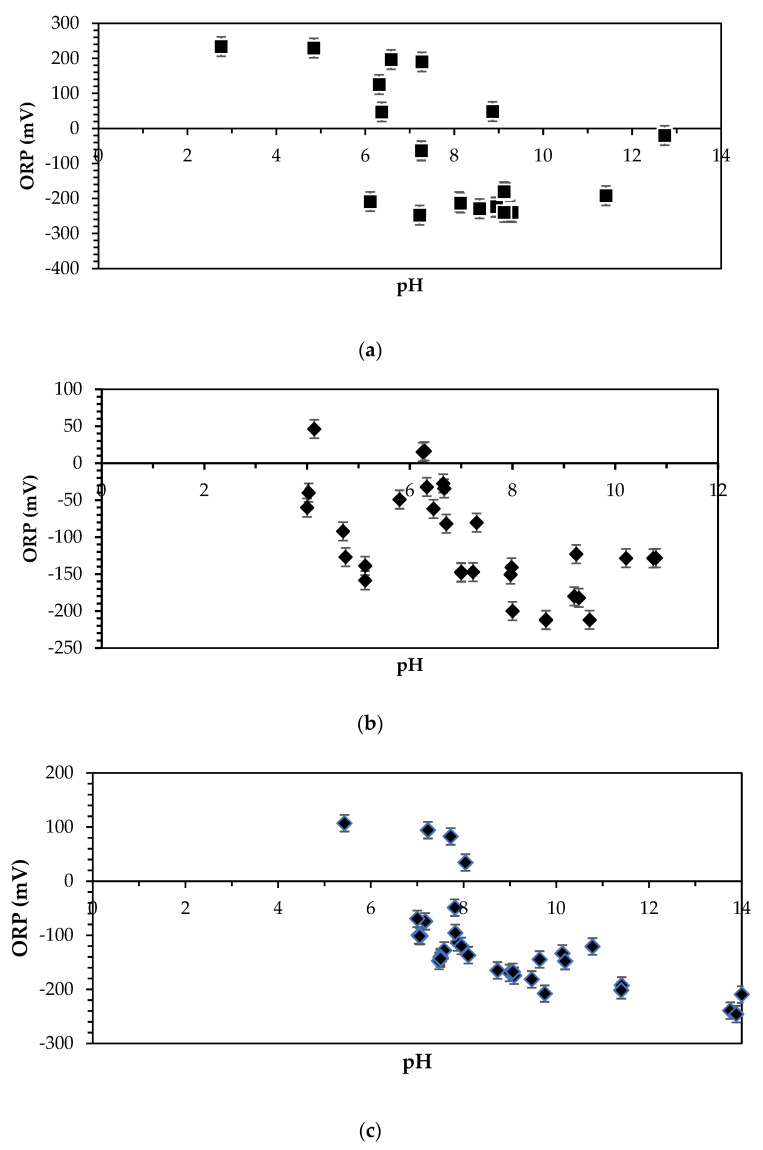
(**a**) ORP vs. pH for Clover wastewater. (**b**) TDS vs. Salinity for biorefinery wastewater. (**c**) ORP vs. pH for mixed wastewater.

Khumalo et al. [42] emphasises the crucial role of oxidation-reduction potential (ORP) in determining the electrical conductivity of wastewater, which is pivotal for identifying suitable electron donors or anolyte streams in bioelectrochemical systems (BES) for bioelectric energy generation. Additionally, Khumalo et al. [27] elaborate on the correlation between pH and ORP, indicating the significance of pH levels within the context of conductivity and electron donation [29,30,31,43,44]. The findings presented in Figure 7a–c confirm that all the investigated wastewater streams exhibit favourable electrical conductivity properties, aligning with established thresholds for viable electron donors in MFC technology [19,27,29,31,43,44,45]. Notably, the optimal pH range for these wastewater streams was observed to be between 6 and 10, accompanied by corresponding ORP values ranging from −300 to −50. This pH-ORP combination signifies an ideal electro-potential conducive to effective electron donation, which is essential for electrical generation in BES and MFC units [27,28].

#### 3.2.4. Taxonomic Classification of Electrical Conductivity (EC) Profiles for All Wastewater Streams

The conductivity of water is influenced by temperature, as indicated by thermodynamic principles [21]. Temperature has a direct impact on the solubility constant of a solution, affecting the overall solubility of minerals present [44,45]. This relationship is particularly relevant in the context of the three distinct wastewater sources examined in this study. An increase in temperature generally enhances solubility, hence positively influencing the conductivity of the liquid [27,32,45,46]. It is important to note that the conductivity of wastewater, which is closely linked to its total dissolved solids, varies across different temperature ranges, such as psychrophilic, mesophilic, or thermophilic conditions [27,33]. Future investigations will explore the specific effects of psychrophilic and mesophilic temperature regimes on electricity production in Double Chamber Microbial Fuel Cells (DCMFCs), utilising Proteobacteria and Bacteriodota as biocatalysts. These studies aim to provide comprehensive insights into this phenomenon, building upon the existing literature [19,20,21]. Elevated conductivity levels are commonly observed at higher temperatures [19].

**Figure 8:**
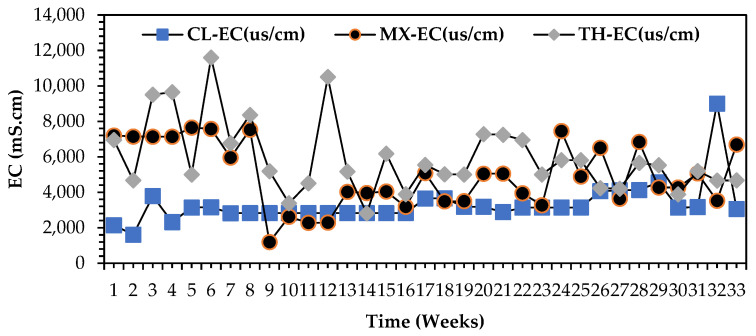
Electrical conductivity comparison plots, all streams.

Based on the findings presented in the Figure 8 comparisons graph for electrical conductivity (EC) for all organic wastewater streams, biorefinery exudes the most viable capacities of all streams considering these vital electrochemical parameters imperative as a perfect bioelectrochemical generation source in the DCMFC unit. The precise range, respectively, for EC for dairy, biorefinery, and mixed wastewater stream chronologically were: 1300 ≤ EC ≤ 8000 mS·cm^2^; 4000 ≤ EC ≤ 12,200 mS·cm^2^. The salinity levels, as presented in the above sections, also favoured high electrochemical potentials of the biorefinery wastewater stream hence validating its feasibility as a recommended anolyte for reliable bioelectricity generation in a DCMFC in this study, [1,2,10,12,13,19,21].

#### 3.2.5. Comparison of the Present Study Wastewater Classification Profiles of Other Studies

Dairy wastewater composition is commonly milk processing effluents that have an increased temperature and large variations in pH, TSS, biological oxygen demand (BOD), chemical oxygen demand (COD), total nitrogen (TN), total phosphorus (TP), and fat, oil, and grease (FOG) [4,5,6,7,8,9]. There is little information on industrial-scale dairy effluent composition, [9]. The information on the general characteristics of dairy wastewater, biorefinery wastewater and mixed wastewater stream is shown in Table S4 in the Supplementary section. Typically, dairy wastewater is white in colour (whey is yellowish green) and has an unpleasant odour and turbid character [2,10,27]. With annual temperatures of 17–25 °C, dairy waste streams are warmer than municipal wastewater (10–20 °C) [7,8], which results in faster biological degradation compared to sewage treatment plants [28]. The average temperatures of industrial dairy effluents range from 17 to 18 °C in winter and 22–25 °C in summer [6]. Using the Arrhenius model, as in the literature, the biodegradation rates and oxygen consumption can be predicted to be 1.5 times higher in summer than in winter [6,7,8,9]. The design winter temperature of 15 °C is adopted for this type of wastewater due to the utilisation of hot water for washing and cleaning of equipment [6,7,8,9,10,11,12,13,17,18,22,40].

In the sugar industry, water is used for cleaning purposes in the different sections of the factory generates wastewater [12,33]. Practically, there are no single units that generate wastewater, but the wastewater is produced mainly by washing on the milling house floor, boiling house like evaporators, clarifiers, vacuum pans, centrifugation [33,34]. Periodic cleaning of lime water and SO₂ production facilities also significantly contributes to the large volume of wastewater, as well as periodic descaling of heat exchangers and evaporators using NaOH, Na_2_CO_3_, and HCl for descaling of heater and neutralisation [33]. Precisely, mill houses, and process houses are the two main sections of wastewater generated in sugar factories [33]. The mill house wastewater is polluted mainly with oil, grease, and suspended solids; whereas the wastewater generated from the process house is contaminated with high organic matter such as COD, BOD_5_, and pH [12,33]. Studies of physicochemical properties of the sugar industrial effluent, dairy industrial effluent, and the mixed stream industrial effluent has been sampled and collected from local wastewater treatment plants and analysed as presented in the supplementary summative Table S4. The detailed values of both physicochemical and organic parameters indicated that the effluent pollutant qualities and quantities are quite different [12,33]. Precisely, looking at the common organic real time process plant data monitoring parameters that were investigated to characterise the wastewater pollutant strength in this study, listing the few critical ones: COD, BOD, TOC, EC, and ORP for the current studied streams, comparatively to previous authors work, the same range of results has been attained. The results align with expectations based on reputable literature [12,33], with mixed wastewater streams presenting high COD and BOD levels due to their organic content [33]. TOC analyses were unique to this study, since most researchers rely on COD for real-time monitoring of wastewater treatment plant performance. The biodegradability ratios of COD: BOD, respectively, closely related for the current study streams: 1.66 ± 1 and previous authors with 1.66 and 1.85, respectively. This current study also thoroughly covered the characteristic physico parameters to present the bioelectrochemical capabilities of these streams. That has been thoroughly digressed in the previous sections and clearly recommended for BES processes application especially in the generation of electricity in MFC processes.

### 3.3. Taxonomy on Biomass rDNA Sequencing and Analysis of Phylum’s Class as Viable Bioelectrochemical Inoculate, After Wastewater Treament Palnt Harvest

#### 3.3.1. Biorefinery (Tongaat Hullet) Biomass–Phylum Classification Blueprint for Sugar Biorefinery Biomass from Wastewater Treatment Plant

This section of the manuscript contains the metagenomics analysis of full-length 16s gene amplicons. As aforementioned, samples were sequenced on the Sequel system by PacBio (www.pacb.com). Raw sub-reads were processed through the SMRTlink (v11.0) Circular Consensus Sequences (CCS) algorithm to produce highly accurate reads (>QV40). These highly accurate reads were processed through DADA2 (https://benjjneb.github.io/dada2/index.html) and qiime2 (https://docs.qiime2.org/2021.11/) for quality control assessment and taxonomic classification, respectively. Create_vsearch_single_sample_pdf_report_pacbio.py 0732022_M13_bc1004_F-M13_bc1068_R.hifi_reads-filtered-feature-table-asv.tsv-M13_bc1004_FM13_bc1068_R0732022 220705_Cell1 16S-QIMME2. Table S1 presents the full layout of the Top phylum family classification of the Biorefinery biomass sample.

#### 3.3.2. Biorefinery (Sugar Mill) Biomass Taxonomical Graphical Classifications

**Figure 9:**
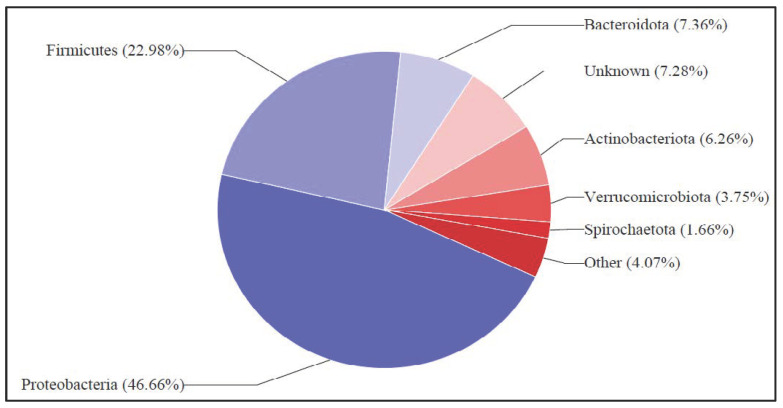
Top-phylum taxonomy classification for a biorefinery wastewater stream.

The detailed genomic analysis was carried out through a series of taxonomical sequences as prelisted: Top-Phylum; Top-Class Classification; Top-Order Classification; Top-Family Classification then finally the Top-Genus Classification on Inqaba Biotec full-length 16s metagenomics report—Sample 3_CloverBiomass_0732022 as presented in Figure 9. The top phylum graphical layout of the bacterial colony’s proportions in the biorefinery sample which correlates with Table S4 (Supplementary Material) was considered for bacterial colonies taxonomical classification. Organisms are grouped into taxa (singular: taxon), and these groups are given a taxonomic rank; groups of a given rank can be aggregated to form a more inclusive group of higher rank, thus creating a taxonomic hierarchy. Precisely, the taxonomy of this bacterial consortium is based on the top phylum graphical classification of the bacterial population, which correlates with the Table S1 family phylum classification, as attached in the Supplementary Materials, which shows a wide margin dominance of Proteobacteria by a read count index ratio of 46.66%. Firmicutes and Bacteriodota follow up sequentially at 22.98% and 7.36%, respectively. A precise observation is that Proteobacteria the dominant species has been applicable in some bio-electrochemical technologies reported by Hossain et al. [35]. Firmicutes phylum is historically related to Geobacter [11,13] and Bacteroidetes biologically and is based on its genomic and rDNA analysis, as reported by [1,34,35,36,37,47,48,49]. This species has been widely reported as a viable electrogene in the generation of bioelectricity in MFCs and other METs, as reported by Logan et al. [36,37,50,51,52]. Firmicutes and Bacteriodota have also been reported by Logan et al. [34,48,52,53] as a good source of electrons or viable anolytes and are efficient for high-strength organic biodegradation and good biological catalyst for the production bioelectricity in an MFC technology.

Automatic identification of the characteristic peaks in the Energy Dispersive X-ray Spectrum (EDS) is a valuable software tool that has been progressively developed with the rise in computing power and speed [35,36,48,49]. The EDS is modern sophistication tool, commonly used for commercial automatic peak identification procedures that are frequently used in the labelling of high intensity peaks that correspond to major constituents [36,48,49]. Based on the Supplementary Material images presented by Figures S2–S4; the EDS pick analysis precisely corresponds to the SEM scans and images that relate a smooth biomass sample with visible patches of white and milky way phases in both scan images, Figure S2. However, the scale bar can be used to possibly identify microstructures and microorganisms, based on the overall size. These SEM images precisely on the red circled areas relates to the EDS peaks analysis that conveys a low peak energy dispersive analysis on the overall biomass sample content, as presented in Figure S2. The lack of calcium on this biorefinery biomass sample will not inhibit the overall process of being a carbon enriched source to facilitate the organic pollutants biodegradation in a typical BES, in the MFC system, referenced by Figure S3.

The energy content peaks of carbon in both samples 1 and 2 images shot up to more than 25 ≤ cps/ev ≤ 40 and an average of 51 wt. % of the mass composition out of 100% total sample population, which makes it a mass fraction of 0.51 (kg·C/Kg·Biomass·Sample), as presented in Figure S4. A basic scientific hypothesis further relating to Figure S4 articulates that the elemental mapping of a biomass sample using Energy Dispersive X-ray Spectroscopy (EDS) for various key elements: Ca, O, C, S, Mg, K, and Cu. These elements are crucial indicators of the bioelectrochemical efficiency of biomass samples, particularly for Microbial Fuel Cell (MFC) applications. Calcium and magnesium are essential for microbial growth and biofilm formation, which enhance microbial activity in MFCs. Their presence suggests that the biomass supports microbial colonisation and stability. The oxygen mapping indicates potential electron acceptor sites or oxidative environments. However, in anaerobic regions (typical for MFC anodic chambers), oxygen levels would need to be limited for effective electron transfer [36,37,47,48,49].

According to Logan et al. [54] and Sarkar et al. [4,5,6,7,8,9], the abundant presence of carbon shows the availability of organic matter, which is vital as an electron donor. Microorganisms can metabolise this carbon to produce electrons for bioelectricity generation in MFCs. Sulphur compounds are often involved in microbial metabolism, particularly for bacteria capable of sulphur reduction. This enhances the variety of metabolic pathways and can contribute to electron donation. Potassium is a vital element for maintaining cellular functions, while copper can act as a catalyst in electron transport processes. The distribution of these elements suggests that the biomass may also provide favourable conditions for electron transfer reactions.

In terms of the biorefinery sample viability, the elemental distribution, especially carbon, sulphur, and other nutrients, indicates that the biomass is rich in organic matter and supportive of microbial metabolic activity. These properties enhance the biomass’s capacity as an electron donor in an MFC, thus improving its bioelectrochemical performance. This analysis implies that the biomass sample could serve as a viable feedstock for sustainable energy generation via microbial-driven processes in MFC systems.

#### 3.3.3. Dairy (Clover) Biomass—Phylum Classification and Taxonomical Blueprint, After Harvest from Wastewater Treatment Plant

Furthermore, this section of the article summarises the metagenomic analysis of full-length 16s gene amplicons of the biomass samples that have been clinically disseminated as per the previous section—biorefinery genomic analysis. Samples were sequenced on the Sequel system by PacBio. Raw sub-reads were processed through the SMRTlink (v11.0) Circular Consensus Sequences (CCS) algorithm to produce highly accurate reads (>QV40). These highly accurate reads were processed through DADA2 (https://benjjneb.github.io/dada2/index.html) and qiime2 (https://docs.qiime2.org/2021.11/) for quality control assessment and taxonomic classification, respectively. create_vsearch_single_sample_pdf_report_pacbio.py-3_Clover_Biomass_1432022_M13_bc1004_F--M13_bc1070_R.hifi_reads-filtered-feature-table-asv.tsv-M13_bc1004_F--M13_bc1070_R 3_Clover_Biomass_1432022 220705_Cell1 16S-QIMME2. Table S4 shows the thorough phylum classification of the dairy biomass. This genomic analysis precisely presents the phylum taxonomical array of the heterotrophic bacterial species, a precise read count, and its ratio analysis.

**Figure 10:**
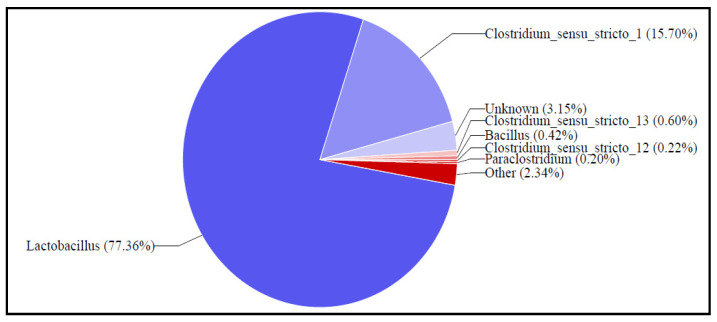
Top-Genus taxonomical classification for dairy wastewater stream.

Sample 1432022, full-length 16s metagonomical report by Inqaba Biotec laboratories:

The taxo-classification of the dairy wastewater bacterial colonies was carried out through a various classification hierarchy as prelisted: The Top-phylum classification was based on Inqaba Biotec full-length 16s metagenomics report—Sample 3_Clover_Biomass_1432022; the Top-Class Classification was based on Inqaba Biotec full-length 16s metagenomics report—Sample 3_Clover_Biomass_1432022; the Top-Order Classification was based on Inqaba Biotec full-length 16s metagenomics report—Sample 3_Clover_Biomass_1432022; the Top-Family Classification, then finally the Top-Genus Classification on Inqaba Biotec full-length 16s metagenomics report—Sample 3_Clover Biomass_1432022. For the simplicity and reliability of this study, the top-phylum taxonomy was considered to analyse the bacterial dominance of the dairy wastewater harvested biomass sample, as presented above in Figure 10. Also, Table S5 in the supplementary section presents the top phylum classification of the bacterial population, which correlates with the graphical family phylum classification, as seen in the figure below. A precise observation that the Lactobacillus bacterial species comprising classification counts and 77.36% of the read counts index was the most dominant bacterial species for this family population [17]. Sequentially and second to the hierarchy of bacterial dominance is Clostridium, at 15.70%. The chloroflexi phylum is vastly related to anaerolineceae, as reported by Liang et al. [54]. Anaerolineceae phylum comprise obligate anaerobes, as a majority in alkali-degrading bacterial colonies, as stated by Logan et al. [54]. Anearolineacea may be associated with the anaerobic degradation of oil-related compounds—this bacterial lineage was also reported as the most frequently encountered bacteria taxon in anaerobic degradation [54,55]. Lactobacillus has been reported by Sarkar et al. [4,5,6,7,8,9] as a common species in the dairy wastewater solid waste and effluent system. A concise understanding is based on the background and origin of the dairy sample [7,9]. Dairy waste organic matter and suspended solid masses essential disintegrates to forms these actively classified biomass genus [3,4,6,17]. Since it is a dairy based sample, it will have to be lactose based and, therefore, the Lactobacillus proved dominant in the metagenomic genus dairy based sample [8,9].

#### 3.3.4. Dairy Biomass Samples 1 and 2 FEG SEM-EDX Analysis via Zeiss Ultra

A separate significant issue is the reliability of elemental identification in the EDS [35,36,48,49]. Automatic identification of the characteristic peaks in the Energy Dispersive X-ray Spectrum (EDS) is a valuable software tool that has been progressively developed with the rise in computing power and speed [36]. The EDS is a modern sophistication tool, commonly used for commercial automatic peak identification procedures that are frequently used in the labelling of high intensity peaks that correspond to major constituents [36,48,49]. Based on the Supplementary Material images, as presented by Figures S5–S7 (Supplementary Section); precisely the EDS pick analysis corresponds to the SEM scans and images that relate a smooth biomass sample with visible patches of white and milky way phases in both scan images, Figure S7. However, the scale bar can be used to possibly identify microstructures and microorganisms, based on the overall size. SEM images highlight circled areas corresponding to EDS peak analysis, indicating low peak energy dispersive values for the overall biomass sample, as shown in Figure S5. Figure S6 intricately presents SEM images of two dairy based samples (Clover 1 and Clover 2) with highlighted areas representing notable surface structures. These structures can be associated with the biofilm formation and microbial colonisation critical for bioelectrochemical activity in Microbial Fuel Cells (MFCs).

The observed surface roughness and specific textures likely indicate regions where microorganisms may anchor, enhancing electron transfer processes between the microbial community and the anode. Such biofilm formations are integral to efficient energy generation in MFCs. The variations in surface characteristics between dairy biomass sample 1 and 2 could imply differences in microbial adhesion or electron transfer efficiency, potentially affecting their performance as MFC inoculants [36]. Further investigation of these samples could confirm their suitability for bioelectrochemical applications, particularly in wastewater treatment and renewable energy generation. The energy content peaks of carbon in both sample 1 and 2 images shot up to more than 40 cps/ev and more than 77% mass composition out of 100% total sample population which makes it a mass fraction of 0.77 (kg·C/Kg·Biomass·Sample), as presented in Figure S7.

#### 3.3.5. Comparison of the Three-Wastewater Substrates and Their Dominant Phylum for DCMFC Treatment and Production of Bioelectricity

**Figure 11:**
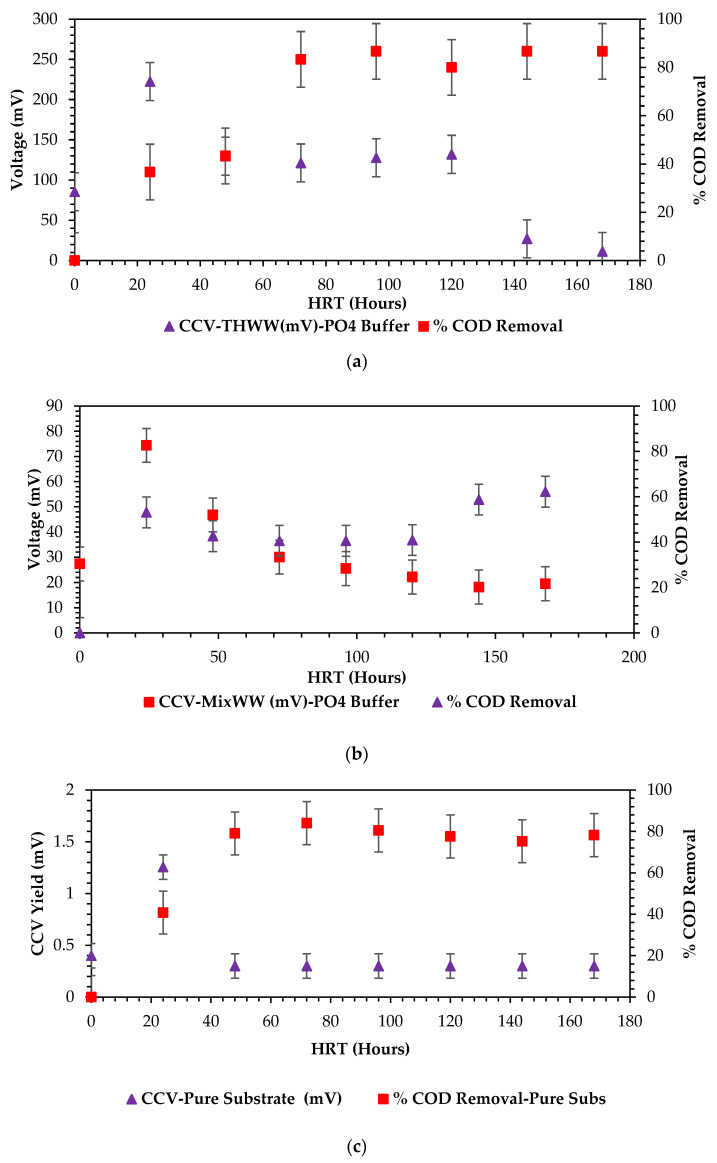
CCV—yield comparisons between all three-streams. (**a**) Biorefinery stream. (**b**) Mixed wastewater stream. (**c**) Dairy wastewater stream, Shabangu et al. [70,71].

Ensuring discharged industrial wastewater meets the required quality standards relies on effective treatment and advanced dewatering technologies, e.g., the BES technology; the MFC system has proven reliability, sustainability, and renewability towards the efficiency of high strength complex industrial substrates and contaminants subsequently generating bioelectricity [24,56,57,58]. Microbial Fuel Cells (MFC) research is a rapidly evolving niche area of research that needs reliable and scalable commercial voltage yield generation from this unit. It is still tricky to compare devices on an equivalent basis [58,59,60,61,62]. Proper operation of this unit requires knowledge of various scientific, wet chemistry, microbial studies, and essentially electro-engineering aspects to be fine-tuned towards scaling up voltage yield generation [23,42,61,63,64,65]. This study has clearly outlined the need to identify the most reliable and readily available raw wastewater sample as a perfect anolyte for the system. More-so, a need for perfectly cultured and active microbes to facilitate the biodegradation aspects of disintegrating chemical organic energy into electrical energy has been raised [61,66,67,68,69]. In view of the taxonomical classification sourced from the comparative analysis of organic substrate in the start-up sequence for double chamber Microbial Fuel Cell (DCMFC) sourced from Shabangu et al. [70,71,72,73]. Figure 11a–c demonstrates intricately the three wastewater streams concurrent to the dissemination of the sRNA and rDNA identification, the subsequent conclusions have been reached; the biorefinery wastewater exudes a massive conductivity capacity, as it shoots up conductivity to around 12,000 uS·cm^2^ and is the most saline raw water source. An overall voltage yield of about 230 mV was reached from pure and raw biorefinery wastewater. The mixed stream source comes second in reliability of conductivity and salinity but exuded the highest recorded voltage yield of 76 mV from pure substrates. Clover wastewater was at the bottom of the taxonomical classification with as low as 1–5 mV generation from a purely raw anolyte feed coupled with the highest phosphates contained compared to all sources. The high phosphates contained explains the inhibition of the bioelectricity generation process [18,40,74,75,76].

Based on the findings of Figure 11a–c, sourced from Shabangu et al. [73,74], biorefinery and mixed wastewater streams are highly recommendable for consideration as reliable anolyte and inoculum in the operation of a typical lab scale unit using purely raw industrial wastewater as a source of electrogeneses and anolyte. The heterotrophs identified and classified in the morphology section states that Protobacteria and Bacteroidetes are recommended as a viable source of electrons when operating the benchtop MFC unit in the subsequent experiments of this study. Biorefinery presented reliability, renewability, and sustainability in terms of being the MFC voltage source supply. Moreso, an organic removal of biodegradable contaminants profile was also shown in Figure 11a–c. A clear depiction is that in all three of the different wastewater sources. The overall percentage removal was achieved within 72 hours of treatment incubation in the MDC unit. Complete 100% removal was observed with mixed wastewater substrates within 72 h of the treatment time. From a scientific point of view, in particular, a bioelectrochemical and wet chemistry or biological perspective, the longer high percentage organic removal is a function of longer incubation periods even in the MFC unit, which relates positively with convectional traditional wastewater treatment strategies. Logan et al. [14,15] and Kim et al. [20] have reported corresponding findings to Shabangu et al. [66,67] in numerous studies they completed in the wastewater treatment capacities using traditional H-shaped Microbial Fuel Cells.

#### 3.3.6. Comparative Summary of Findings of Current Paper vs. Previous Studies on MFCs

Huang et al. [77] report advancements in Microbial Fuel Cell (MFC) technology for detecting organic matter, focusing on biochemical oxygen demand (BOD) and chemical oxygen demand (COD). It was gathered that dual-chamber and single-chamber MFCs are prominent, with single-chamber designs offering simplicity and cost-effectiveness but reduced stability. Emerging designs, such as miniaturised, submersible, and coupled MFCs (e.g., wetland-integrated), have expanded application scenarios and improved detection capabilities. However, limitations include the need for stable microbial communities, resistance to fouling, and interference from toxicants or competing electron acceptors. The study highlights the transition of MFCs from prototypes to practical environmental monitoring tools, emphasising design and operational innovations for scalable, reliable, and self-powered systems. Integration with artificial intelligence is proposed to enhance data interpretation and adaptability.

Loveccho et al. [78] present a customised multichannel measurement system for Microbial Fuel Cell (MFC) characterisation, featuring an expandable design capable of simultaneously measuring up to 12 MFCs. The system includes multi-step discharge protocols, long-term measurement capabilities with variable time steps, and calibration procedures to ensure accurate low-current signal detection. Key contributions include bridging the gap between laboratory-grade equipment and cost-effective, scalable tools for MFC research, with robust calibration and multi-channel capabilities suitable for complex systems. This system has the potential to accelerate advancements in renewable energy and wastewater treatment technologies. Limitations include dependence on rigorous calibration, reliance on simulated testing, and scalability challenges for applications beyond 12 MFCs, requiring specialised hardware and software modifications.

The current research study explores the potential of a Double Chamber Microbial Fuel Cell (DCMFC) for electricity generation using three distinct wastewater sources—biorefinery, dairy, and mixed streams. It emphasises the importance of substrate selection and microbial community composition for optimising bioelectrochemical processes. Key findings highlight that biorefinery wastewater offers the highest bioelectrochemical potential due to superior electrical conductivity and salinity, achieving a voltage yield of 65 mV, compared to 75.2 mV for mixed streams and 1.7 mV for dairy wastewater. Proteobacteria, Firmicutes, and Bacteriodota were identified as dominant phyla in biorefinery samples, while Lactobacillus and Clostridium were prevalent in dairy samples. Limitations include the inhibitory effects of high phosphate levels in dairy streams and challenges in scalability and microbial stability.

As new knowledge contributions, the study bridges the gap between industrial wastewater characterisation and bioelectricity generation, leveraging metagenomics to identify suitable microbial consortia. It establishes correlations between wastewater physicochemical properties (e.g., salinity, conductivity) and energy output, advancing MFC scalability and reliability. Lastly, this study proposes biorefinery wastewater as a viable source for sustainable electricity production and efficient substrate removal.

Furthermore, the findings support the use of MFCs in wastewater treatment and renewable, sustainable and reliable bioenergy generation. Challenges such as optimising microbial stability, scalability for viable commercial application, and overcoming substrate-specific limitations like high phosphate interference, specifically in the dairy wastewater organic substrate as an anolyte, are clearly highlighted. Moreover, this work underpins the foundation for future advancements in commercial scale bioelectrochemical systems. The following comparative Table 1 lays the key concepts from the current study comparatively to previous work.

## 4. Conclusions and Recommendations

### 4.1. Conclusions

In the quest to ensure compliance with stringent industrial wastewater quality standards, the adoption of effective treatment and advanced dewatering technologies is imperative. Among these technologies, the Bioelectrochemical System (BES), specifically the Microbial Fuel Cells (MFCs) system, has exhibited remarkable reliability, sustainability, and renewability in addressing the challenges posed by high-strength complex industrial substrates and contaminants, ultimately yielding bioelectricity [24,56,57,58]. The domain of Microbial Fuel Cells (MFCs) is rapidly evolving and demands the development of reliable and scalable commercial voltage generation units [58,59,60,61,62]. Achieving optimal MFC operation necessitates a multifaceted approach encompassing scientific knowledge, wet chemistry, microbial studies, and electro-engineering aspects, all tailored toward enhancing voltage yield generation [23,42,61,63,64,65]. This study underscores the critical importance of identifying the most dependable and readily available raw wastewater samples for use as anolytes within the MFC system. Moreover, the necessity for well-cultured and active microbial consortia to facilitate the biodegradation of complex organic compounds into electrical energy has been underscored [61,66,67,68,69]. Based on the taxonomical classification, along with the concurrent analysis of small RNA (sRNA) and ribosomal DNA (rDNA) identification from the three complex wastewater sources, the following conclusions have emerged: Biorefinery wastewater exhibits substantial conductivity capacity, elevating conductivity levels to approximately 12,000 µS·cm, making it the most saline raw water source. It yielded an overall voltage of approximately 230 mV when processed from pure and untreated biorefinery wastewater. The mixed stream wastewater source ranks second in terms of conductivity and salinity reliability. Remarkably, it recorded the highest voltage yield of 76 mV when processed from pure substrates. Clover wastewater, on the other hand, is situated at the lower end of the taxonomical classification, yielding a mere 1–5 mV when subjected to raw anolyte feeding. This result is correlated with its notably high phosphate content, which appears to inhibit the bioelectricity generation process, [18,40,74,75,76]. These findings highlight the potential of different wastewater sources for bioelectricity generation in MFCs, with biorefinery and mixed stream wastewater sources showing promise for further exploration and optimisation in scaling up voltage yield generation.

### 4.2. Recommendations

Based on the findings presented in Figure 11a,b, sourced from Shabangu et al. [70,71,72,73] several important recommendations have been drawn:

i. Biorefinery and mixed wastewater streams emerge as highly viable options for serving as reliable anolyte and inoculum sources in operating this benchtop DCMFC unit utilising purely raw industrial wastewater as an electrogenic bacterial community and efficient anolyte.
ii. The heterotrophs classified in the morphology section, specifically Proteobacteria and Bacteroidetes, are considered as viable sources of electrons for operating this benchtop DCMFC unit in subsequent experiments of this study. Biorefinery wastewater stands out for its reliability, renewability, and sustainability in terms of being the primary DCMFC bioelectricity source.
iii. Organic removal or biodegradable contaminants as shown in Figure 11a, b, demonstrated that in all three different wastewater sources, the overall percentage removal was achieved within a short span of time, specifically, within 72 h of treatment incubation in the DCMFC technology. Complete 100% removal was observed with mixed wastewater substrates within the same 72 h treatment period.

From a scientific perspective, precisely in the field of bioelectrochemistry, wet chemistry, and biodegradation principles, it is evident that the duration of high-percentage organic removal is influenced by the length of incubation periods, in the DCMFC unit. This finding of this study primarily relates and validates previous work on conventional wastewater treatment strategies reported in studies conducted by Shabangu et al. [70,71,72,73], Logan et al., [14,15] and Kim et al. [20] using traditional H-shaped Microbial Fuel Cells. In summary, the choice of biorefinery and mixed wastewater streams as anolyte sources in MFC technology and the identification of Proteobacteria and Bacteroidetes as viable sources of electrons are key takeaways from this study, emphasising the efficiency and rapidity of organic removal within MFC units. These findings contribute to innovating the field of industrial wastewater treatment and bioelectrochemical processes for practicality and applicability at commercial scale platforms.

### 4.3. Future Perspectives

Based on the above underpinned findings and conclusions of this study, the following future directions can be highlighted:

i. Expansion of genetically engineered microorganisms for tailored applications.
ii. Real-time signal processing improvements with MATLAB/SIMSCAPE-simulated empirical algorithms.
iii. Focus on optimising the bioenergy capacity of the MFC unit via integration with power boosting electrical components towards meeting the national grid connection IEEE standards for the practicality and applicability of this technology in fighting the current energy and freshwater scarcity in South Africa.

This paper establishes DCMFC-organic pollutant-based unit as a promising technology for environmental pollution monitoring and treatment while generating bioelectricity as a multifaceted approach. It highlights the need for ongoing optimisation to address current challenges commercial scale bioenergy production and practical application.

## Figures and Tables

**Table 1:** Comparative summary of findings: current vs. previous MFC studies.

Category	Doble Chamber Microbial Fuel Cell-Based on Industrial Organic Matter: Current Study	Customised Multichannel Measurement System:	Critical Findings in MFC-Based Organic Sensors:
		Loveccho et al. [78]	Huang et al. [77]
Key Findings	- MFC sensors enable BOD and COD detection via microbial metabolism.	- Developed a multi-channel, portable system for real-time MFC monitoring.	- Comprehensive review of various MFC sensor designs and their applications.
	- Advantages: real-time, low-cost, rapid monitoring.	- Accurate measurement of voltage, current, and power with ±10% error.	- Types include dual-chamber, single-chamber, miniaturised, and submersible MFCs.
	- Types: Double chamber, coupled MFC systems.	- Expandable for up to 12 MFCs and customizable protocols.	- Optimisation via electrode materials, microbial strains, and real-time algorithms.
	- Novel-electrode configuration like CCu-3 (Carbon-Copper) enhance bioelectrochemical capacity.	- Validated with simulated MFCs, but live system validation is pending.	- Identifies key performance factors (e.g., pH, temperature, flow rate).
Optimisation	- Improved sensitivity with CCu-based electrodes.	- Calibration ensures accuracy but requires external expertise.	- Emphasises integration with AI for better signal processing and adaptability.
	- Enhanced designs improve stability and reduce oxygen leakage.	- Modular hardware allows scalability but limited to 12 MFCs without redesign.	- Multi-stage MFCs improve detection range and toxic interference resistance.
Applications	- Adaptable for domestic, industrial, and synthetic wastewater systems.	- Provides insights for MFC optimisation in renewable energy and wastewater treatment.	- Proven for monitoring diverse wastewater types, including coupled wetland systems.
Challenges	- Biomass fouling, and enhanced metabolism issues.	- Dependence on rigorous calibration and lack of live-system testing.	- Challenges include maintaining microbial community stability and high costs of complex systems.
Contributions	- Proposes transition of MFCs from prototypes to practical monitoring BES for grid power connection.	- Bridges gap between high-cost lab tools and scalable solutions for MFC research.	- Proposes genetically engineered microbes and AI-driven optimisation as future directions.
Future Directions	- Focus on grid standards power generation, automation, and solar powered DCMFC designs.	- Integration of data analytics for in-depth performance optimisation.	- Expanding detection capabilities and scalability for real-world environmental monitoring.

## Data Availability

Data are available on request from the authors of this study.

## Supplementary Materials

The following supporting information can be downloaded at: https://www.mdpi.com/article/10.3390/bioengineering12010088/s1, Table S1, Welch two-sample student *t*-test method and 95% Confidence Level comparison between all wastewater streams on the organic/chemical parameters; Table S2, Welch two-sample student *t*-test method and 95% Confidence Level comparison between all wastewater streams for the physico parameters; Table S3, Analysis of variation (ANOVA) test method for Tukey mean averages and 95% Confidence Level family wise; Table S4, Classification results industrial wastewater pollutants, current and previous studies; Table S5, Top Phylum Classification Biorefinery Biomass Morphology; Table S6, Top Phylum Classification for Dairy Biomass Morphology; Figure S1, Salinity vs Resistivity for all WW streams: (a) Dairy Wastewater substrates (b) Biorefinery Wastewater substrates (c) Mixed Wastewater substrates; Figure S2, Images on Biorefinery Biomass samples 1 and 2 via Zeiss Ultra and FEG SEM-EDX; Figure S3, Biorefinery biomass sigma component elements weight percentage layout done via the energy dispersive X-Ray unit (EDX) with detailed elemental analysis and sigma ratios. Elemental, beam energy (cps/eV), showing correct identification of the C, O, Mg, S, K, Ca, and Cu elements that are characterised in the dairy biomass sample. Cu element seems to be misidentified at the end of the peak energy curves; Figure S4, Component Light images by Nikon Eclipse 80i Compound Fluorescent Light microscope for Tongaat Hullet Biomass samples 1 and 2. The respective scale bar can be used to possibly identify microstructures & microorganisms, based on the overall size. This Scans presents the cross-section view of the bacterial sample. C-Blue, O-Red, S-Green, Mg-Pink, Ca-Purple, K-dark Blue and Cu-dark Green; Figure S5, Images on Clover Biomass samples 1 and 2 via Zeiss Ultra and FEG SEM-EDX； Figure S6， Clover Dairy elemental, beam energy (cps/eV), showing correct identification of the C, O, Mg, S, K, Ca, and Cu elements that are characterised in the dairy biomass sample. Cu elementseems to be misidentified at the end of the peak energy curves; Figure S7, Component Light images by Nikon Eclipse 80i Compound Fluorescent Light microscope for Clover-Dairy Biomass samples 1 and 2. The respective scale bar can be used to possibly identify microstructures & microorganisms, based on the overall size. This Scans presents the cross-section view of the bacterial sample. C-Blue, O-Red, S-Green, Mg-Pink, Ca-Purple, K-dark Blue and Cu-dark Green.
