# Supplementary material for: Metagenomic Insights into Pollutants in Biorefinery and Dairy Wastewater: rDNA Dominance and Electricity Generation in Double Chamber Microbial Fuel Cells
Source: Bioengineering (Basel). 2025 Jan 19;12(1):88. doi: 10.3390/bioengineering12010088 (PMC11761944; doi:10.3390/bioengineering12010088)
Supplement: Supplementary file 1 [file bioengineering-12-00088-s001.zip › bioengineering-3379659-supplementary.pdf]

bioengineering

MDPI

Supplementary Materials

Table S1. Welch two-sample student t-test method and 95% Confidence Level comparison between all wastewater streams on the organic / chemical parameters.

TOTAL ORGANIC CARBON: TOC (mgTOC/L)			
Parameters	*Stream Sources*		
	TH-CL	TH-MX	CL-MX
**t-value**	-1,741	-2,905	-1,991
**difference**	23,05	18,909	24,165
**alpha / p-value**	0,095	0,00909	0,0579
**95% CI**	(-333,5 to 28,7)	(-986,3 to -160,2)	(-856,8 to 15,2)
**mean values**	176,4 - 328,8	176,4 - 749,6	328,8 - 749,7
**COV: value**		1,39	
**CARBON OXYGEN DEMAND: COD (mgCOD/L)**			
Parameters	*Stream Sources*		
	TH-CL	TH-MX	CL-MX
**t-value**	-1,437	-2,934	-2,14
**difference**	22,96	18,9	24,216
**alpha / p-value**	0,164	0,00854	0,0426
**95% CI**	(-941,6 to 169,7)	(-3033,5 to -507,2)	(-2718,6 to -50,1)
**mean values**	492,6 - 878,5	492,6 - 2262,9	878,6 - 2262,9
**COV: value**		1,476	
**TOTAL PHOSPHATES: PO4 (mgPO$_4$/L)**			
Parameters	*Stream Sources*		
	TH-CL	TH-MX	CL-MX
**t-value**	-8,641	-4,33	-0,0778
**difference**	19,32	18,31	26,008
**alpha / p-value**	4,58E-08	0,000389	0,939
**95% CI**	(-375,9 to -229,5)	(-458,4 to -159,2)	(-168,3 to 156,0)
**mean values**	67,4 - 370,1	67,4 - 376,2	370 - 376,2
**COV: value**		0,899	

Table S2. Welch two-sample student t-test method and 95% Confidence Level comparison between all wastewater streams for the physico parameters.

SALINITY: (ppt SAL)			
Parameters	*Stream Sources*		
	TH-CL	TH-MX	CL-MX
**t-value**	4,663	2,105	-3,413
**difference**	54,9	53,69	63,9
**alpha / p-value**	2,03E-05	0,0399	0,0011

**95% CI**	(0,9366 to2,349)	(0,0349 to 1,434)	(-1,441 to -0,377)
**mean values**	3,424 - 1,782	3,424 - 2,690	3,424 - 2,690
**COV: value**		0,558	
**DISSOLVED OXYGEN: DO (mgDO/L)**			
Parameters	*Stream Sources*		
	**TH-CL**	**TH-MX**	**CL-MX**
**t-value**	7,63	-1,52	-11,23
**difference**	36,59	62,11	38,49
**alpha / p-value**	4,42E-09	0,133	1,02E-13
**95% CI**	(15,006 to 25,854)	(-11,87 to 1,61)	(-30,17 to -20,96)
**mean values**	27,57 - 7,14	27,57 - 32,71	7,14 - 32,72
**COV: value**		0,704	
**ELECTRICAL CONDUCTIVITY: EC (mS/cm²)**			
Parameters	*Stream Sources*		
	**TH-CL**	**TH-MX**	**CL-MX**
**t-value**	-8,641	2,043	-4,35
**difference**	19,32	63,29	54,35
**alpha / p-value**	4,58E-08	0,0453	6,07E-05
**95% CI**	(-375,9 to -229,5)	(21,38 to 1940,56)	(-2420,2 to -8892,9)
**mean values**	67,4 - 370,1	5933,7 - 4952,7	3296,2 - 4952,7
**COV: value**		0,43	
**TOTAL DISSOLVED SOLIDS: (mgTDS/L)**			
Parameters	*Stream Sources*		
	**TH-CL**	**TH-MX**	**CL-MX**
**t-value**	4,663	2,37	-3,36
**difference**	54,9	55,29	63,99
**alpha / p-value**	2,03E-05	0,0213	0,00135
**95% CI**	(0,9366 to2,349)	(107,3 to 1279,2)	(-1219,1 to -308,6)
**mean values**	3,424 - 1,782	3169,5 - 2476,3	1712,5 - 2476,3
**COV: value**		0,5	
**RESISTIVITY: MOhms**			
Parameters	*Stream Sources*		
	**TH-CL**	**TH-MX**	**CL-MX**
**t-value**	-7,12	-1,63	3,46
**difference**	59,86	47,69	55,9
**alpha / p-value**	1,57E-09	0,109	1,03E-03
**95% CI**	(-1,9E-04 to 1,06E-04)	(-1,01E-04 to 1,03E-05)	(4,34E-04 to 1,63E-04)
**mean values**	1,81E-04 - 3,3E-04	(1,81E-04 - 2,2E-04	3,3E-04 - 2,27E-04
**COV: value**		0,49	

Table S3. Analysis of variation (ANOVA) test method for Tukey mean averages and 95% Confidence Level family wise.

SALINITY: (ppt SAL)			
Parameters	*Stream Sources*		
	**CL-TH**	**MX-TH**	**MX-CL**
**difference**	-1,64	-0,733	0,909
**lower**	-2,42	-1,506	0,135
**upper**	-8,69E-01	0,0396	1,682
**p-adje**	0,0000061	0,0667	0,0169
**DISSOLVED OXYGEN: DO (mgDO/L)**			
Parameters	*Stream Sources*		
	**CL-TH**	**MX-TH**	**MX-CL**
**difference**	-20,43	5,13	25,56
**lower**	-27,12	-1,56	18,87
**upper**	-1,37E+01	11,83	3,23E+01
**p-adje**	0	0,166	0
**ELECTRICAL CONDUCTIVITY: EC (mS/cm²)**			
Parameters	*Stream Sources*		
	**CL-TH**	**MX-TH**	**MX-CL**
**difference**	-2637,5	-980,9	1656,5
**lower**	-3652,4	-1995,9	641,6
**upper**	-1,62E+03	33,9	2,67E+03
**p-adje**	0	0,06	0,00054
**TOTAL DISSOLVED SOLIDS: (mgTDS/L)**			
Parameters	*Stream Sources*		
	**CL-TH**	**MX-TH**	**MX-CL**
**difference**	-1457,1	-693,3	763,8
**lower**	-2106,5	-1342,7	114,4
**upper**	-8,08E+02	-43,8	1413,3
**p-adje**	0	0,033	0,016
**RESISTIVITY: MOhms**			
Parameters	*Stream Sources*		
	**CL-TH**	**MX-TH**	**MX-CL**
**difference**	1,48E-04	4,54E-05	-1,03E-04
**lower**	0,0000856	-0,0000174	-0,000165
**upper**	2,11E-04	1,08E-04	-4,02E-05
**p-adje**	0,0000005	0,202	0,000515

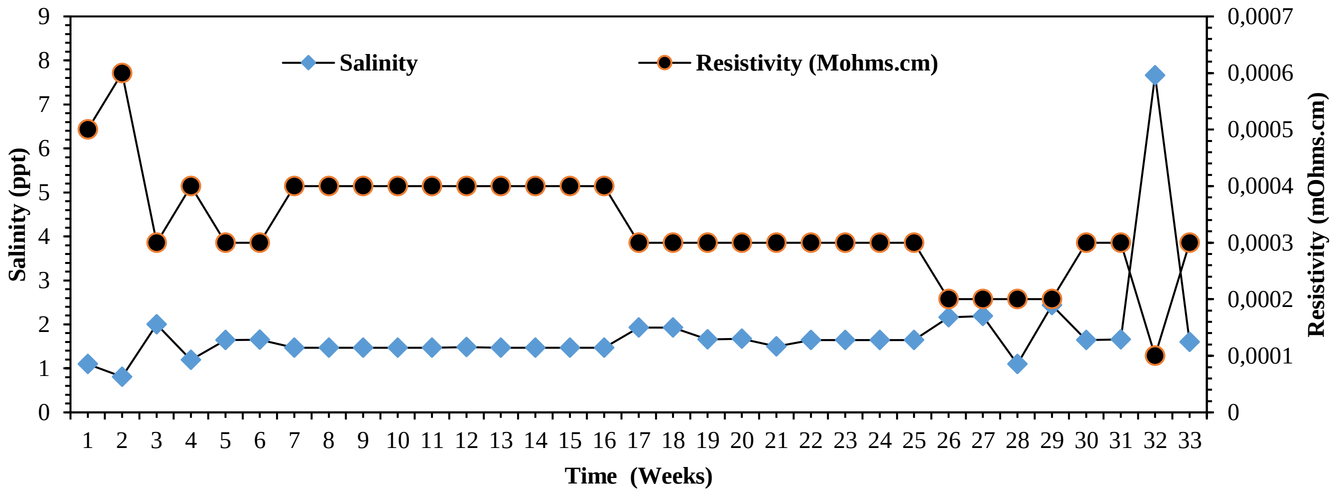
Salinity
Resistivity (Mohms.cm)
Salinity (ppt)
Resistivity (mOhms.cm)
Time (Weeks)

(a)

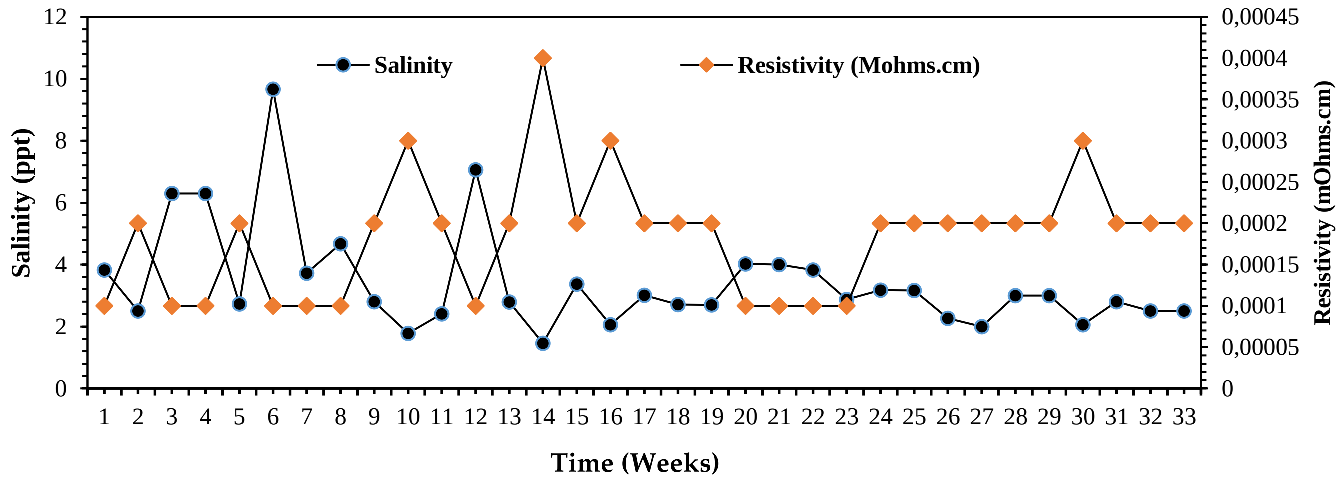
Salinity
Resistivity (Mohms.cm)
Salinity (ppt)
Resistivity (mOhms.cm)
Time (Weeks)

(b)

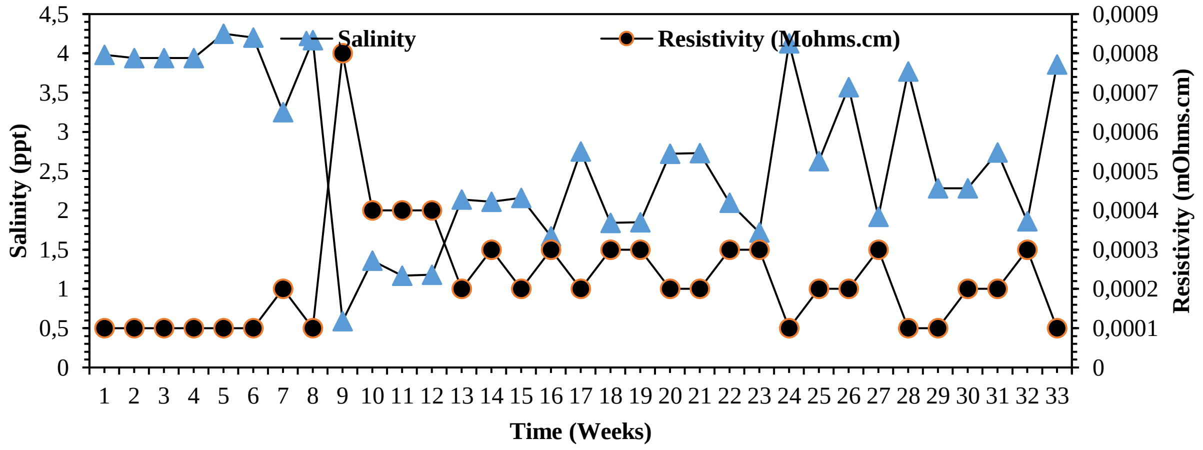
Salinity
Resistivity (Mohms.cm)
Salinity (ppt)
Resistivity (mOhms.cm)
Time (Weeks)

(c)

Figure S1. Salinity vs Resistivity for all WW streams: **(a)** Dairy Wastewater substrates **(a)** Biorefinery Wastewater substrates **(c)** Mixed Wastewater substrates.

Comparison of the Current Study Wastewater Classification Profile With Previous Research Work.

Table S4. Classification results industrial wastewater pollutants, current and previous studies.

**Constituent**	**Clover-WW** Current Study	**Biorefinery-WW** Current Study	**Mixed-WW** Current Study	**Dairy-WW** [7–9]	**Bioref-WW** [47]	**Mixed-WW** [4–6,9]
Active reaction - pH	2,76-12,73	4-10,79	5,43-14,00	9,1 ± 6,7	7,5 - 8	4 - 11
Temperature °C	18-25	17-24	18-26	17 - 25	22 - 25	22 - 25
COD (Chemical Oxygen Demand) mg/L	128-3455	65-1870	152-9965	2040 -4730	45,000 – 52,000	500 – 10400
TOC mg/L	63-1168,1	38-639,7	45,2-4070	-	-	-
BOD (Biological Oxygen Demand) mg/L	768-2073	39-1122	91,2-5979	1080 -2810	8 000 – 10 000	240 – 5900
COD / BOD ratio	1.667	1,665	1,666	1,68	5,2	1,85
VFA (Volatile Fatty Acids) mg/L	-	-	-	240 - 290	-	20 – 1920
PO_4^{-3} (Total-Phosphates) mg/L	75-674,5	13,40-161,0	36-1100	20 - 30	1500 - 1700	0 – 600
TKN (Total Kjedahl Nitrogen) mg/L	-	-	-	-	4000 - 4200	10 – 660
TS (Total Solids) mg/L	-	-	-	-	70,000 – 75,000	710 - 7000
Turbidity NTU	59-2800	26-400	48-1866	-	-	-
TSS (Total Suspended Solids) mg/L	202 - 9580	89 - 1369	165 - 6385	530 - 1130	38,000 – 42,000	60 – 580
TDS (Total Dissolved Solids) mg/L	804-6626	1405-8212	595-3824	-	30,000 – 32,000	-
EC (Electrical Conductivity) µS.cm	13,25-4562	2810-10550	1191-7647	-	-	-
Salinity ppt	0,81-7,66	1,46-9,66	0,59-4,25	-	-	-
Resistivity Ω	0,0004	0,0002	0,0002	-	-	-
DO mg/L	2,99-20,28	5,05-45,06	11,51-47,03	-	-	-
pHmV	-359,8-57,30	-261,7-97,7	-428,5-19	-	-	-
Oxidation Reduction Potential (ORP)	-247,6-233,7	-212-46,30	-245,8-107,2	-	-	-

Table S5. Top Phylum Classification Biorefinery Biomass Morphology.

**Phyla Classification**	**Read Count**	**%**
Proteobacteria	1744	46,66
Firmicutes	859	22,98
Bacteroidota	275	7,36
Unknown	272	7,28
Actinobacteriota	234	6,26
Verrucomicrobiota	140	3,75
Spirochaetota	62	1,66
Patescibacteria	28	0,75
Synergistota	23	0,62
Planctomycetota	22	0,59
Desulfobacterota	17	0,45
Chloroflexi	17	0,45
Hydrogenedentes	16	0,43
Deferribacterota	9	0,24
Dependentiae	8	0,21
Armatimonadota	2	0,05
Cloacimonadota	2	0,05
Campilobacterota	2	0,05
Sumerlaeota	2	0,05
Fermentibacterota	2	0,05
Marinimicrobia	1	0,03
SAR324_clade	1	0,03

Table S6. Top Phylum Classification for Dairy Biomass Morphology.

**Genus Classification**	**Read Count**	**%**
Lactobacillus	3464.0	77.36
Clostridium_sensu_stricto_	1 703.0	15.70
Unknown	141.0	3.15
Clostridium_sensu_stricto_	13 27.0	0.60
Bacillus	19.0	0.42
Clostridium_sensu_stricto_	12 10.0	0.22
Paraclostridium	9.0	0.20

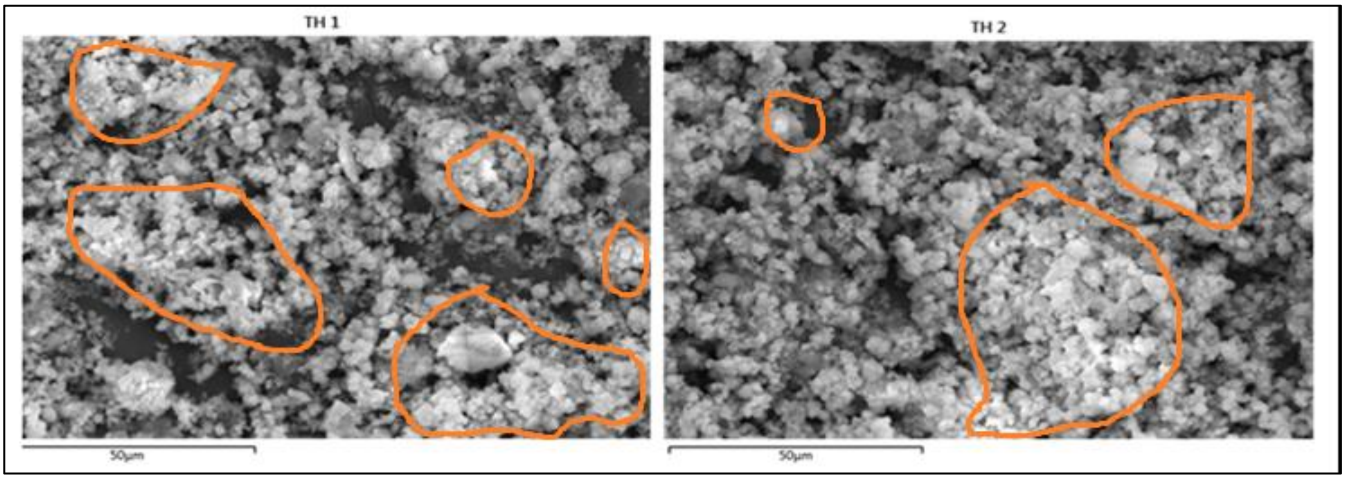

Figure S2. Images on Biorefinery Biomass samples 1 and 2 via Zeiss Ultra and FEG SEM- EDX.

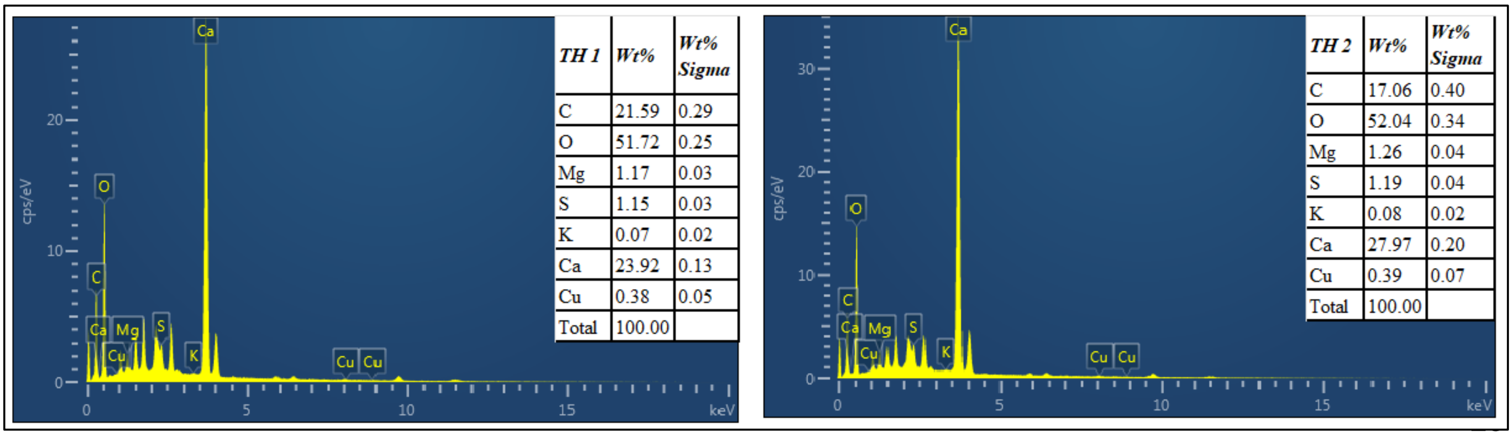

TH 1	*Wt%*	*Wt% Sigma*
C	21.59	0.29
O	51.72	0.25
Mg	1.17	0.03
S	1.15	0.03
K	0.07	0.02
Ca	23.92	0.13
Cu	0.38	0.05
Total	100.00	

TH 2	*Wt%*	*Wt% Sigma*
C	17.06	0.40
O	52.04	0.34
Mg	1.26	0.04
S	1.19	0.04
K	0.08	0.02
Ca	27.97	0.20
Cu	0.39	0.07
Total	100.00	

Figure S3. Biorefinery biomass sigma component elements weight percentage layout done via the energy dispersive X-Ray unit (EDX) with detailed elemental analysis and sigma ratios. Elemental, beam energy (cps/eV), showing correct identification of the C, O, Mg, S, K, Ca, and Cu elements that are characterised in the dairy biomass sample. Cu element seems to be misidentified at the end of the peak energy curves.

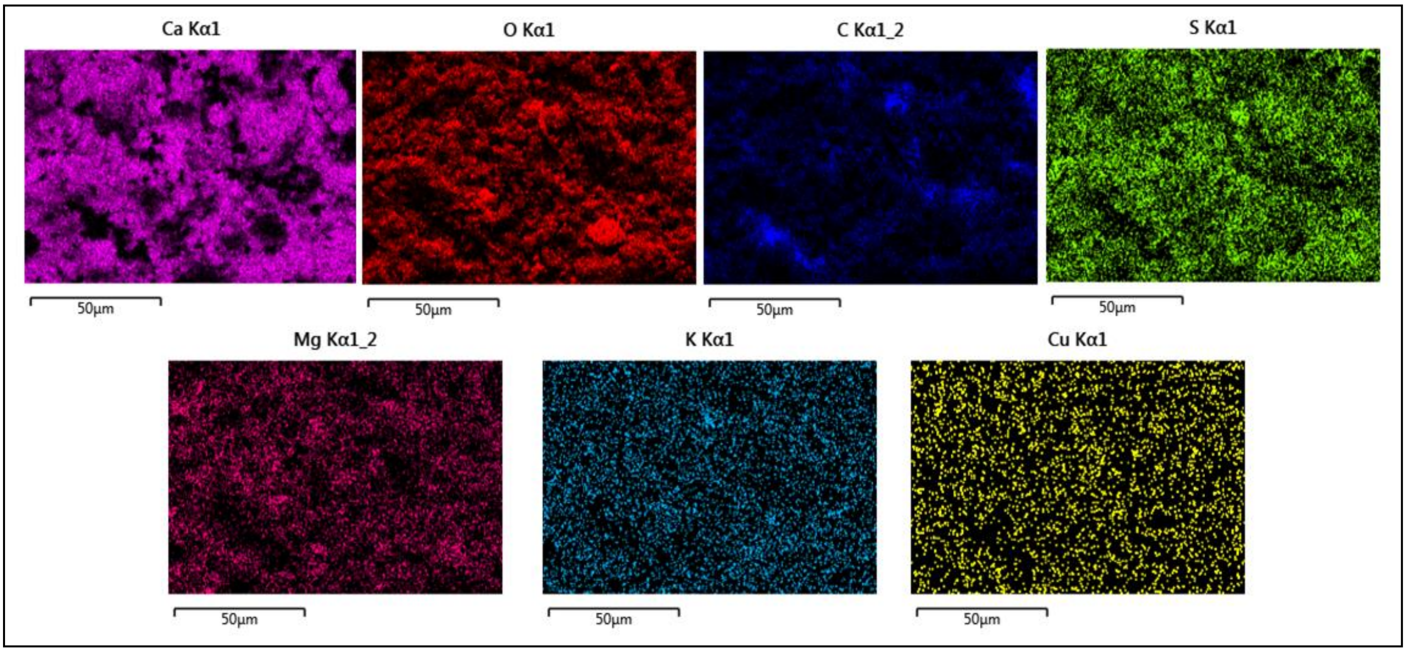

Figure S4. Component Light images by Nikon Eclipse 80i Compound Fluorescent Light microscope for Tongaat Hullet Biomass samples 1 and 2. The respective scale bar can be used to possibly identify microstructures & microorganisms, based on the overall size. This Scans presents the cross-section view of the bacterial sample. C-Blue, O-Red, S-Green, Mg-Pink, Ca-Purple, K-dark Blue and Cu-dark Green.

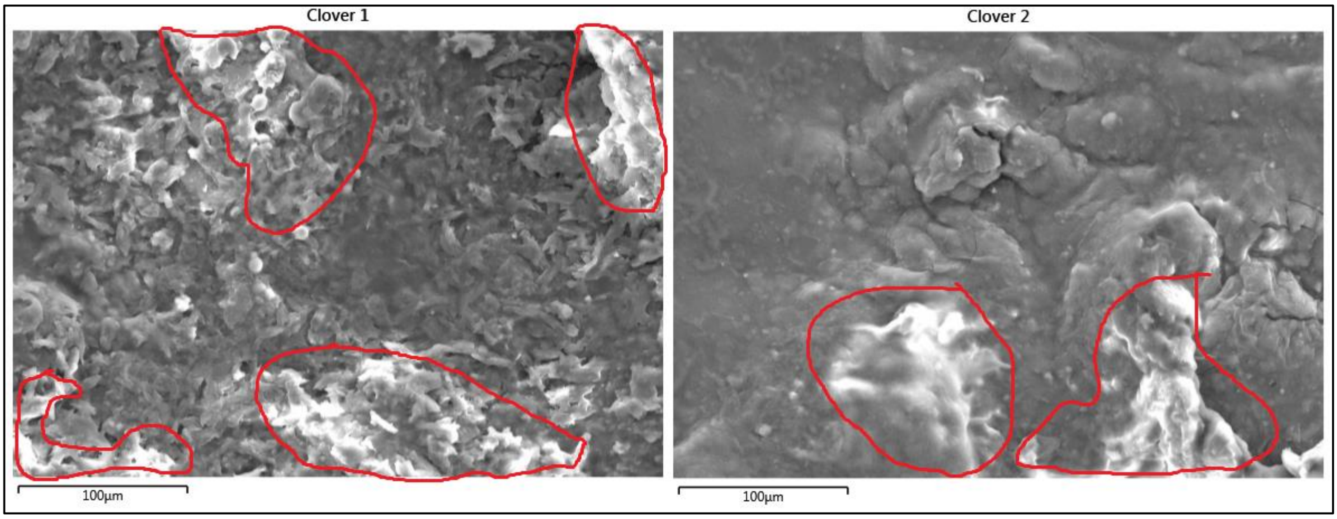

Figure S5. Images on Clover Biomass samples 1 and 2 via Zeiss Ultra and FEG SEM- EDX.

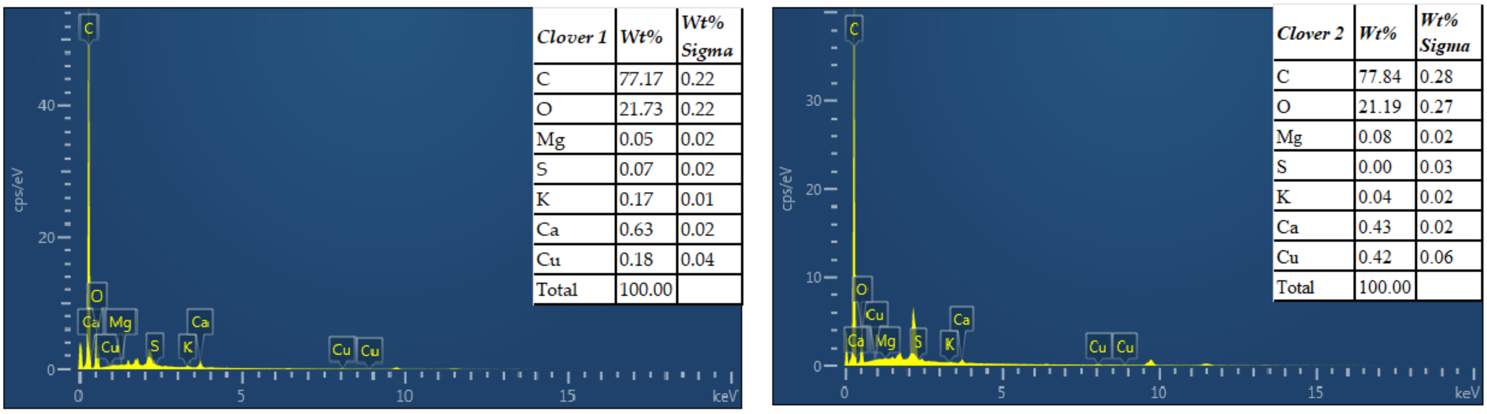

Clover 1	*Wt%*	*Wt% Sigma*
C	77.17	0.22
O	21.73	0.22
Mg	0.05	0.02
S	0.07	0.02
K	0.17	0.01
Ca	0.63	0.02
Cu	0.18	0.04
Total	100.00	

Clover 2	*Wt%*	*Wt% Sigma*
C	77.84	0.28
O	21.19	0.27
Mg	0.08	0.02
S	0.00	0.03
K	0.04	0.02
Ca	0.43	0.02
Cu	0.42	0.06
Total	100.00	

Figure S6. Clover Dairy elemental, beam energy (cps/eV), showing correct identification of the C, O, Mg, S, K, Ca, and Cu elements that are characterised in the dairy biomass sample. Cu element seems to be misidentified at the end of the peak energy curves.

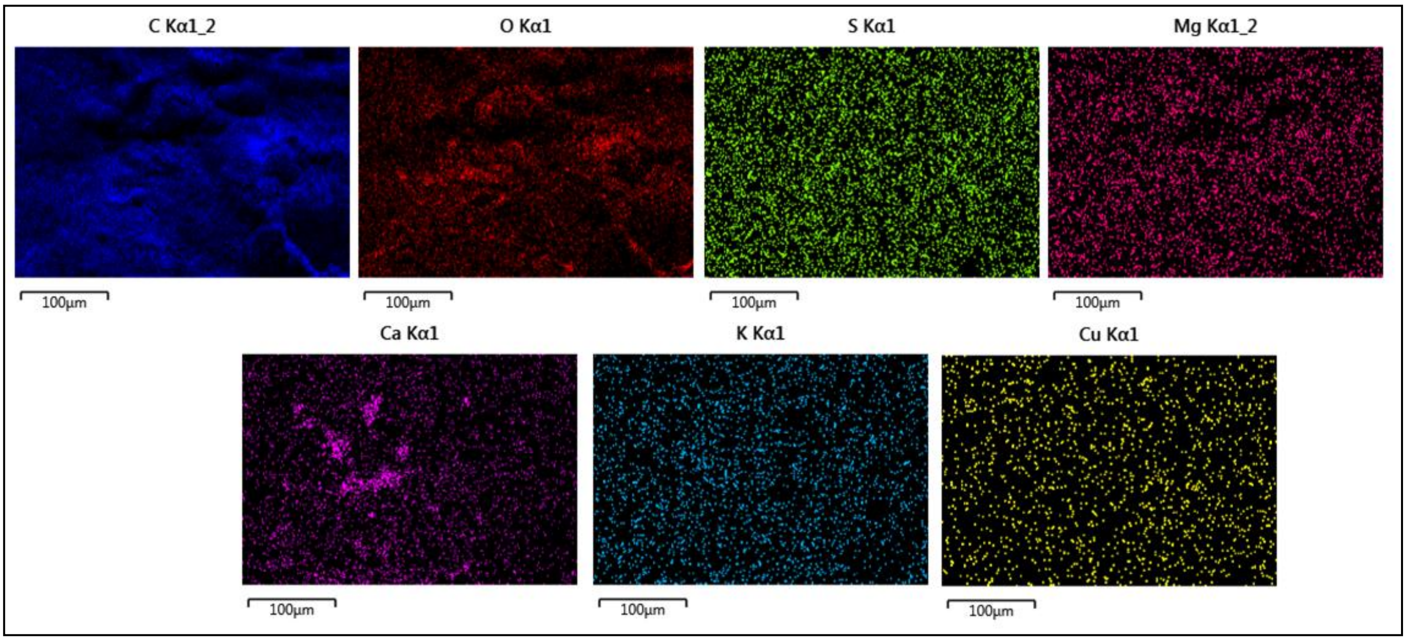

Figure S7. Component Light images by Nikon Eclipse 80i Compound Fluorescent Light microscope for Clover-Dairy Biomass samples 1 and 2. The respective scale bar can be used to possibly identify microstructures & microorganisms, based on the overall size. This Scans presents the cross-section view of the bacterial sample. C-Blue, O-Red, S-Green, Mg-Pink, Ca-Purple, K-dark Blue and Cu-dark Green.
